# Correction: Relative Abundance of and Composition within Fungal Orders Differ between Cheatgrass (*Bromus tectorum*) and Sagebrush (*Artemisia tridentata*)-Associated Soils

**DOI:** 10.1371/journal.pone.0123849

**Published:** 2015-03-30

**Authors:** The PLOS ONE Staff

## Notice of Republication

This article was republished on March 12, 2015, to correct a spelling error in the title that was introduced during the production process. The publisher apologizes for the error. Please download this article again to view the correct version. The originally published, uncorrected article and the republished, corrected article are provided here for reference.

## Supporting Information

S1 File Originally published, uncorrected article. (PDF)

S2 File Republished, corrected article. (PDF)
