# Supplementary figures and images for: Relative Abundance of and Composition within Fungal Orders Differ between Cheatgrass (Bromus tectorum) and Sagebrush (Artemisia tridentata)-Associated Soils
Source: PLoS One. 2015 Jan 28;10(1):e0117026. doi: 10.1371/journal.pone.0117026 (PMC4309613; doi:10.1371/journal.pone.0117026)

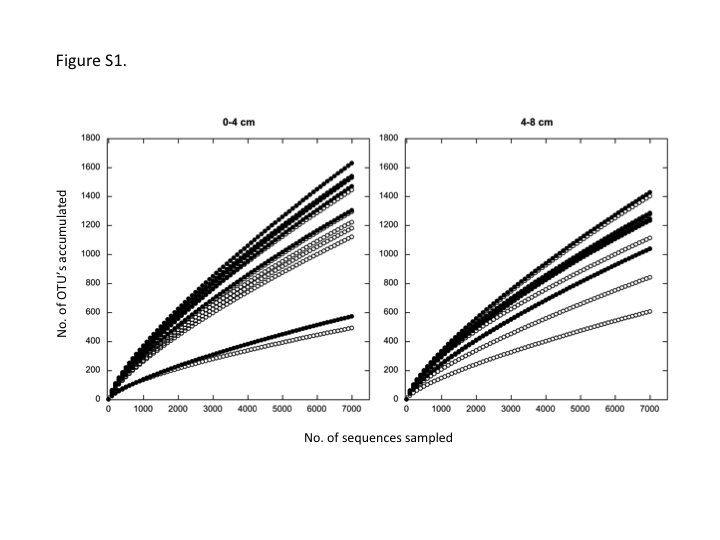

Supplement: S1 Fig — OTU's defined at a maximum distance of 0.03. Left panel contains rarefaction curves for the 0–4 cm depth intervals and right panel contains rarefaction curves from the 4–8 cm depth intervals. Sagebrush soils (●); cheatgrass soils (◯). (TIFF) [file pone.0117026.s001.tiff]

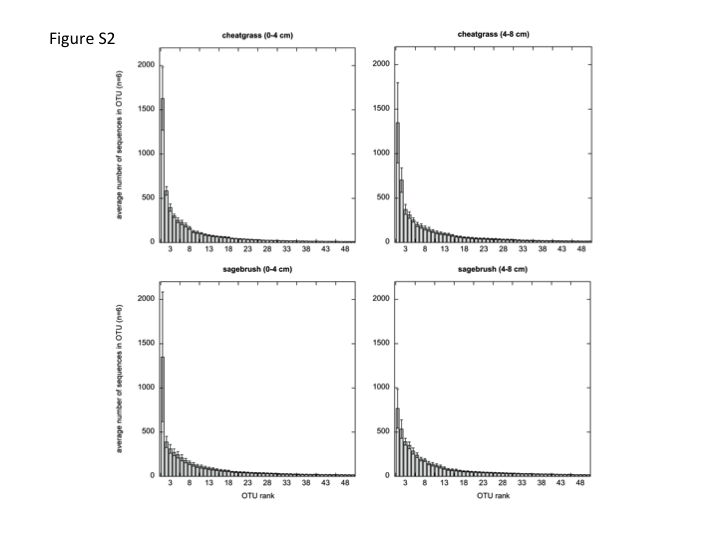

Supplement: S2 Fig — Data for each rank is the average number of sequences in that rank (n = 6 standard error). (TIFF) [file pone.0117026.s002.tiff]
