# Supplementary material for: Relative Abundance of and Composition within Fungal Orders Differ between Cheatgrass (Bromus tectorum) and Sagebrush (Artemisia tridentata)-Associated Soils
Source: PLoS One. 2015 Jan 28;10(1):e0117026. doi: 10.1371/journal.pone.0117026 (PMC4309613; doi:10.1371/journal.pone.0117026)
Supplement: S1 Table — (DOCX) [file pone.0117026.s003.docx]

**Table S1.**

| **Sequence library** | **total seqs.** | **fungi (100%)** | **phylum (80%)** | **class (80%)** | **order (80%)** |
| --- | --- | --- | --- | --- | --- |
| **C4T** | 14889 | 14594 | 14571 | 13622 | 13121 |
| **C5T** | 12805 | 12259 | 12218 | 10546 | 10086 |
| **C6T** | 16202 | 16083 | 16040 | 15409 | 14699 |
| **C7T** | 15057 | 14871 | 14813 | 14160 | 13655 |
| **C8T** | 12889 | 12777 | 12725 | 11954 | 11495 |
| **C9T** | 20640 | 20402 | 20402 | 20061 | 19734 |
| **C4B** | 21002 | 20673 | 20638 | 20312 | 18660 |
| **C5B** | 7037 | 5171 | 5027 | 4793 | 4265 |
| **C6B** | 11133 | 10893 | 10841 | 10375 | 8603 |
| **C7B** | 15245 | 12525 | 12402 | 12103 | 9820 |
| **C8B** | 14783 | 14666 | 14655 | 14504 | 13203 |
| **C9B** | 7810 | 7630 | 7614 | 7349 | 6138 |
| **S4T** | 17263 | 16412 | 16345 | 15472 | 12586 |
| **S5T** | 10253 | 9867 | 9672 | 9227 | 7851 |
| **S6T** | 11747 | 11529 | 11482 | 10943 | 10209 |
| **S7T** | 10042 | 9805 | 9742 | 8566 | 8050 |
| **S8T** | 18102 | 18029 | 17962 | 17791 | 17268 |
| **S9T** | 7592 | 7433 | 7366 | 6918 | 6592 |
| **S4B** | 18023 | 17373 | 17202 | 16744 | 16453 |
| **S5B** | 12863 | 8591 | 8492 | 8307 | 7460 |
| **S6B** | 7835 | 6535 | 6335 | 5994 | 5706 |
| **S7B** | 18827 | 14174 | 13837 | 12895 | 11790 |
| **S8B** | 13038 | 10525 | 10269 | 10143 | 9962 |
| **S9B** | 17970 | 15119 | 14924 | 14343 | 12853 |
| **Sequence Library Summary** | | |  |  |  |
| **average** | 13876.95833 | 12830.66667 | 12732.25 | 12188.79167 | 11260.79167 |
| **range** | 7037 to 21002 | 5117 to 20673 | 5027 to 20638 | 4793 to 20312 | 4265 to 19734 |
| **stdev** | 4160.601278 | 4217.750458 | 4238.725077 | 4225.763702 | 4152.179626 |
| **total** | 333047 | 307936 | 305574 | 292531 | 270259 |
