# Supplementary material for: Relative Abundance of and Composition within Fungal Orders Differ between Cheatgrass (Bromus tectorum) and Sagebrush (Artemisia tridentata)-Associated Soils
Source: PLoS One. 2015 Jan 28;10(1):e0117026. doi: 10.1371/journal.pone.0117026 (PMC4309613; doi:10.1371/journal.pone.0117026)
Supplement: S2 Table — Composite libraries for each of the four soil intervals include the libraries generated from the six field replicates. (DOCX) [file pone.0117026.s004.docx]

**Table S2.**

| **Composition of Sequences Classifying at the Genus-Level within the** | | | | |
| --- | --- | --- | --- | --- |
| **Order Agaricales** | |  |  |  |
|  |  |  |  |  |
| **genus** | **CT** | **ST** | **CB** | **SB** |
| Psathyrella |  | 0.822320117 | 0.393030263 | 61.27470754 |
| Macrocystidia | | 0.16886931 | 1.034979693 | 11.05284389 |
| Coprinopsis | 0.43344519 | 0.43318649 | 0.248919167 | 7.139975797 |
| Clitopilus |  | 95.54331865 | 0.039303026 | 5.566760791 |
| Conocybe | 0.629194631 | 0.242290749 | 7.192453819 | 3.186768858 |
| Gastrocybe | 1.062639821 | 0.587371512 | 84.01676929 | 2.258975393 |
| Simocybe |  |  |  | 1.774909238 |
| Lophiostoma | |  |  | 1.53287616 |
| Resupinatus |  |  |  | 1.492537313 |
| Nolanea |  | 0.007342144 | 0.026202018 | 0.927793465 |
| Lycoperdon | 0.006991051 | 1.769456681 |  | 0.927793465 |
| Tulostoma | 0.013982103 | 0.022026432 | 0.065505044 | 0.524405002 |
| Marcelleina |  |  |  | 0.32271077 |
| Tetracladium | |  |  | 0.282371924 |
| Gibberella |  |  |  | 0.242033078 |
| Penicillium |  |  |  | 0.161355385 |
| Arrhenia | 7.270693512 | 0.073421439 | 0.222717149 | 0.121016539 |
| Chaetomidium | |  |  | 0.121016539 |
| Endoperplexa | |  |  | 0.121016539 |
| Eupenicillium | |  |  | 0.121016539 |
| Sclerotinia |  |  |  | 0.080677693 |
| Psilocybe | 0.041946309 |  | 3.773090528 | 0.040338846 |
| Lepiota | 89.6602349 | 0.03671072 | 1.07428272 | 0.040338846 |
| Coprinellus | 0.713087248 | 0.029368576 | 0.96947465 | 0.040338846 |
| Calvatia | 0.006991051 |  |  | 0.040338846 |
| Alternaria |  |  |  | 0.040338846 |
| Aniptodera |  |  |  | 0.040338846 |
| Cercophora |  |  |  | 0.040338846 |
| Coniochaeta |  |  |  | 0.040338846 |
| Cordyceps |  |  |  | 0.040338846 |
| Cyphellophora | |  |  | 0.040338846 |
| Hypocrea |  |  |  | 0.040338846 |
| Lecanicillium |  |  |  | 0.040338846 |
| Montagnula |  |  |  | 0.040338846 |
| Petriella |  |  |  | 0.040338846 |
| Pochonia |  |  |  | 0.040338846 |
| Pyrenochaeta | |  |  | 0.040338846 |
| Saccobolus |  |  |  | 0.040338846 |
| Tilletia |  |  |  | 0.040338846 |
| Tricladium |  |  |  | 0.040338846 |
| Agrocybe | 0.006991051 |  | 0.930171623 |  |
| Volvariella |  | 0.029368576 | 0.013101009 |  |
| Cyathus |  | 0.183553598 |  |  |
| Omphalina |  | 0.029368576 |  |  |
| Agaricus | 0.006991051 | 0.022026432 |  |  |
| Melanoleuca | 0.118847875 |  |  |  |
| Chamaeota | 0.006991051 |  |  |  |
| Delicatula | 0.006991051 |  |  |  |
| Macrolepiota | 0.006991051 |  |  |  |
| Psathyrellaceae incertae sedis | 0.006991051 |  |  |  |
|  |  |  |  |  |
| sequences not classified | 8.583114974 | 27.20470337 | 55.46934251 | 58.31511687 |
|  |  |  |  |  |
| unique genera | 5 | 2 | 0 | 26 |
|  |  |  |  |  |
| genera detected | 17 | 16 | 14 | 40 |
