# Supplementary material for: Relative Abundance of and Composition within Fungal Orders Differ between Cheatgrass (Bromus tectorum) and Sagebrush (Artemisia tridentata)-Associated Soils
Source: PLoS One. 2015 Jan 28;10(1):e0117026. doi: 10.1371/journal.pone.0117026 (PMC4309613; doi:10.1371/journal.pone.0117026)
Supplement: S3 Table — Composite libraries for each of the four soil intervals include the libraries generated from the six field replicates. (DOCX) [file pone.0117026.s005.docx]

**Table S3.**

| **Composition of Sequences Classifying at the Genus-Level within the** | | | | |
| --- | --- | --- | --- | --- |
| **Order Pleosporales** | |  |  |  |
|  |  |  |  |  |
| **Genus** | **CT** | **ST** | **CB** | **SB** |
| Phaeosphaeria | 71.9065 | 39.7724 | 25.9232 |  |
| Alternaria | 20.8242 | 33.1265 | 8.9066 | 4.4413 |
| Murispora | 2.5929 | 4.8616 | 0.3983 |  |
| Westerdykella | 1.2791 | 0.3103 | 0.0112 | 0.0205 |
| Preussia | 0.5788 | 1.7067 | 0.2534 | 0.0409 |
| Karstenula | 0.5498 | 5.0944 | 3.1137 | 1.1052 |
| Stagonosporopsis | 0.3878 |  |  |  |
| Pyrenophora | 0.3531 |  |  |  |
| Stagonospora | 0.3531 |  | 0.1086 |  |
| Camarosporium | 0.2894 | 0.8275 | 0.2534 | 0.0409 |
| Loratospora | 0.2604 | 0.5689 | 0.2896 | 0.0205 |
| Pyrenochaeta | 0.1447 | 2.4308 | 33.0558 | 15.3500 |
| Lophiostoma | 0.1389 | 6.3615 | 6.4084 | 26.6680 |
| Herpotrichia | 0.1331 |  | 3.1137 | 0.0409 |
| Keissleriella | 0.0695 | 2.0171 | 7.7842 | 0.4707 |
| Montagnula | 0.0289 | 0.2327 | 6.8429 | 44.9038 |
| Pleospora | 0.0289 |  | 0.0724 |  |
| Chalastospora | 0.0174 | 0.1552 | 0.1086 |  |
| Entodesmium | 0.0116 | 0.0776 |  |  |
| Drechslera | 0.0116 |  |  |  |
| Ophiosphaerella | 0.0058 | 0.0259 |  | 0.0205 |
| Corynespora | 0.0058 |  | 0.0362 | 0.0205 |
| Chaetosphaeronema | 0.0058 | 0.0776 | 0.0362 |  |
| Massariosphaeria | 0.0058 | 0.0776 |  |  |
| Cochliobolus | 0.0058 | 0.0259 |  |  |
| Delitschia | 0.0058 |  |  |  |
| Phaeodothis | 0.0058 |  | 0.0362 |  |
| Sarcinomyces |  |  |  | 2.9267 |
| Thelebolus |  |  |  | 1.6169 |
| Arrhenia |  |  |  | 0.8596 |
| Byssothecium |  | 0.0259 |  | 0.5731 |
| Rhizophydium |  |  |  | 0.2251 |
| Setomelanomma |  | 0.0259 |  | 0.1023 |
| Marcelleina |  |  |  | 0.1023 |
| Gibberella |  |  |  | 0.0614 |
| Scolecobasidiella |  |  |  | 0.0614 |
| Heleiosa |  |  | 0.3259 | 0.0409 |
| Saccobolus |  |  |  | 0.0409 |
| Cyphellophora |  |  |  | 0.0205 |
| Endoperplexa |  |  |  | 0.0205 |
| Kriegeria |  |  |  | 0.0205 |
| Orbicula |  |  |  | 0.0205 |
| Paecilomyces |  |  |  | 0.0205 |
| Penicillium |  |  |  | 0.0205 |
| Peyronellaea |  |  |  | 0.0205 |
| Sclerotinia |  |  |  | 0.0205 |
| Didymella |  | 2.0171 | 0.0362 |  |
| Leptosphaeria |  | 0.1293 |  |  |
| Edenia |  | 0.0517 |  |  |
|  |  |  |  |  |
| % sequences not | 56.84 | 72.91 | 71.43 | 61.64 |
| classified |  |  |  |  |
|  |  |  |  |  |
| unique genera | 5 | 5 | 1 | 16 |
|  |  |  |  |  |
| genera recovered | 27 | 23 | 21 | 32 |
