# Supplementary material for: Relative Abundance of and Composition within Fungal Orders Differ between Cheatgrass (Bromus tectorum) and Sagebrush (Artemisia tridentata)-Associated Soils
Source: PLoS One. 2015 Jan 28;10(1):e0117026. doi: 10.1371/journal.pone.0117026 (PMC4309613; doi:10.1371/journal.pone.0117026)
Supplement: S4 Table — Composite libraries for each of the four soil intervals include the libraries generated from the six field replicates. (DOCX) [file pone.0117026.s006.docx]

**Table S4.**

| **Composition of Sequences Classifying at the Genus-Level within the** | | | | |
| --- | --- | --- | --- | --- |
| **Order Hypocreales** | |  |  |  |
|  |  |  |  |  |
| **genus** | **CT** | **ST** | **CB** | **SB** |
| Gibberella | 98.7341772 | 36.9146005 | 52.2222222 | 8.35509138 |
| Hydropisphaera | 0.69749418 | 0.13774104 | 38.7037037 | 2.08877284 |
| Hypocrea | 0.28416429 | 33.4710743 | 5.92592592 | 6.26631853 |
| Elaphocordyceps | 0.10333247 |  | 0.09259259 |  |
| Cordyceps | 0.05166623 | 0.13774104 |  | 7.31070496 |
| Emericellopsis | 0.02583311 | 0.27548209 |  | 0.78328981 |
| Neonectria | 0.02583311 |  | 0.83333333 |  |
| Paecilomyces | 0.02583311 | 0.96418732 | 0.92592592 | 1.82767624 |
| Pochonia | 0.02583311 | 16.3911845 | 0.46296296 | 3.13315926 |
| Stachybotrys | 0.02583311 |  |  |  |
| Alternaria |  |  |  | 0.52219321 |
| Aniptodera |  |  |  | 0.26109660 |
| Beauveria |  | 0.13774104 |  |  |
| Cercophora |  |  |  | 0.26109660 |
| Chaetomidium |  |  |  | 1.82767624 |
| Coniochaeta |  |  |  | 0.78328981 |
| Dothidea |  |  |  | 0.26109660 |
| Endoperplexa |  |  |  | 2.34986945 |
| Eupenicillium |  |  |  | 0.78328981 |
| Lecanicillium |  | 7.98898071 | 0.18518518 | 6.52741514 |
| Lophiostoma |  |  |  | 11.7493472 |
| Marcelleina |  |  |  | 3.39425587 |
| Nectria |  |  | 0.64814814 |  |
| Nectriopsis |  | 0.13774104 |  |  |
| Niesslia |  | 3.44352617 |  | 0.26109660 |
| Nolanea |  |  |  | 0.26109660 |
| Orbicula |  |  |  | 0.26109660 |
| Penicillium |  |  |  | 25.8485639 |
| Psilocybe |  |  |  | 0.52219321 |
| Saccobolus |  |  |  | 0.26109660 |
| Sarcinomyces |  |  |  | 0.26109660 |
| Sclerotinia |  |  |  | 0.26109660 |
| Tetracladium |  |  |  | 13.0548302 |
| Tilletia |  |  |  | 0.26109660 |
| Tulostoma |  |  |  | 0.26109660 |
|  |  |  |  |  |
|  |  |  |  |  |
| sequences not classified | 4.655172414 | 40.44298605 | 54.4495993 | 68.00334169 |
|  |  |  |  |  |
| unique genera | 1 | 2 | 1 | 20 |
|  |  |  |  |  |
| genera detected | 10 | 11 | 9 | 29 |
