# Supplementary material for: Relative Abundance of and Composition within Fungal Orders Differ between Cheatgrass (Bromus tectorum) and Sagebrush (Artemisia tridentata)-Associated Soils
Source: PLoS One. 2015 Jan 28;10(1):e0117026. doi: 10.1371/journal.pone.0117026 (PMC4309613; doi:10.1371/journal.pone.0117026)
Supplement: S5 Table — Composite libraries for each of the four soil intervals include the libraries generated from the six field replicates. (DOCX) [file pone.0117026.s007.docx]

**Table S5.**

| **Composition of Sequences Classifying at the Genus-Level within the** | | | | |
| --- | --- | --- | --- | --- |
| **Order Helotiales** | |  |  |  |
|  |  |  |  |  |
| **genus** | **CT** | **ST** | **CB** | **SB** |
| Sclerotinia | 46.93615656 | 6.726246472 | 0.190735695 | 0.109051254 |
| Cudoniella | 43.27879371 | 0.917215428 | 87.14804723 | 0.054525627 |
| Tricladium | 8.950914341 | 7.008466604 | 2.506811989 | 11.94111232 |
| Tetracladium | 0.834135387 | 79.02163688 | 10.14532243 | 61.39585605 |
| Aniptodera |  |  |  | 0.054525627 |
| Botryotinia |  |  | 0.009082652 |  |
| Cercophora |  |  |  | 0.054525627 |
| Chaetomidium |  |  |  | 0.708833152 |
| Chaetomium |  |  |  | 0.054525627 |
| Chalara |  | 6.138287865 |  |  |
| Coniochaeta |  |  |  | 0.163576881 |
| Crinula |  | 0.023518344 |  |  |
| Endoperplexa |  |  |  | 0.381679389 |
| Gibberella |  |  |  | 0.109051254 |
| Hymenoscyphus | | 0.16462841 |  |  |
| Lophiostoma |  |  |  | 1.199563795 |
| Marcelleina |  |  |  | 0.109051254 |
| Orbicula |  |  |  | 2.344601963 |
| Paecilomyces |  |  |  | 0.054525627 |
| Penicillium |  |  |  | 1.035986914 |
| Pochonia |  |  |  | 0.109051254 |
| Pyrenochaeta |  |  |  | 0.054525627 |
| Sarcinomyces |  |  |  | 19.79280262 |
| Tilletia |  |  |  | 0.272628135 |
|  |  |  |  |  |
|  |  |  |  |  |
| sequences not classified | 54.13788035 | 38.63472363 | 11.65850919 | 76.08242045 |
|  |  |  |  |  |
|  |  |  |  |  |
| unique genera | 0 | 3 | 1 | 16 |
|  |  |  |  |  |
| genera detected | 4 | 7 | 5 | 20 |
