# Supplementary material for: Correction: Relative Abundance of and Composition within Fungal Orders Differ between Cheatgrass (Bromus tectorum) and Sagebrush (Artemisia tridentata)-Associated Soils
Source: PLoS One. 2015 Mar 30;10(3):e0123849. doi: 10.1371/journal.pone.0123849 (PMC4378941; doi:10.1371/journal.pone.0123849)
Supplement: S1 File — (PDF) [file pone.0123849.s001.pdf]

PLOS ONE

RESEARCH ARTICLE

Relative Abundance of and Composition within Fungal Orders Differ between Cheatgrass (*Bromus tectorum*) and Sagebrush (*Artemisia tridentate*)-Associated Soils

Carolyn F. Weber[1]*, Gary M. King[2], Ken Aho[1]

1 Department of Biological Sciences, Idaho State University, Pocatello, Idaho, United States of America, **2** Department of Biological Sciences, Louisiana State University, Baton Rouge, Louisiana, United States of America

* webecaro@isu.edu

CrossMark
click for updates

OPEN ACCESS

Citation: Weber CF, King GM, Aho K (2015) Relative Abundance of and Composition within Fungal Orders Differ between Cheatgrass (*Bromus tectorum*) and Sagebrush (*Artemisia tridentate*)-Associated Soils. PLoS ONE 10(1): e0117026. doi:10.1371/journal.pone.0117026

Academic Editor: Matthew Germino, US Geological Survey, UNITED STATES

Received: July 30, 2014

Accepted: December 17, 2014

Published: January 28, 2015

Copyright: © 2015 Weber et al. This is an open access article distributed under the terms of the Creative Commons Attribution License, which permits unrestricted use, distribution, and reproduction in any medium, provided the original author and source are credited.

Data Availability Statement: Sequences have been deposited into MG-RAST (http://metagenomics.anl.gov) under the following ID numbers: 4574367.3, 4574369.3, 4574371.3, 4574373.3, 4574375.3, 4574377.3, 4574379.3, 4574381.3, 4574383.3, 4574385.3, 4574387.3, 4574389.3, 4574366.3, 4574368.3, 4574370.3, 4574372.3, 4574374.3, 4574376.3, 4574378.3, 4574380.3, 4574382.3, 4574384.3, 4574386.3, 4574388.3.

Funding: This project was funded by new faculty start up funding provided by a National Science Foundation EPSCoR grant (EPS-0814387) and new

Abstract

Nonnative *Bromus tectorum* (cheatgrass) is decimating sagebrush steppe, one of the largest ecosystems in the Western United States, and is causing regional-scale shifts in the predominant plant-fungal interactions. Sagebrush, a native perennial, hosts arbuscular mycorrhizal fungi (AMF), whereas cheatgrass, a winter annual, is a relatively poor host of AMF. This shift is likely intertwined with decreased carbon (C)-sequestration in cheatgrass-invaded soils and alterations in overall soil fungal community composition and structure, but the latter remain unresolved. We examined soil fungal communities using high throughput amplicon sequencing (ribosomal large subunit gene) in the 0–4 cm and 4–8 cm depth intervals of six cores from cheatgrass- and six cores from sagebrush-dominated soils. Sagebrush core surfaces (0–4 cm) contained higher nitrogen and total C than cheatgrass core surfaces; these differences mirrored the presence of glomalin related soil proteins (GRSP), which has been associated with AMF activity and increased C-sequestration. Fungal richness was not significantly affected by vegetation type, depth or an interaction of the two factors. However, the relative abundance of seven taxonomic orders was significantly affected by vegetation type or the interaction between vegetation type and depth. Teloschistales, Spizellomycetales, Pezizales and Cantharellales were more abundant in sagebrush libraries and contain mycorrhizal, lichenized and basal lineages of fungi. Only two orders (Coniochaetales and Sordariales), which contain numerous economically important pathogens and opportunistic saprotrophs, were more abundant in cheatgrass libraries. Pleosporales, Agaricales, Helotiales and Hypocreales were most abundant across all libraries, but the number of genera detected within these orders was as much as 29 times lower in cheatgrass relative to sagebrush libraries. These compositional differences between fungal communities associated with cheatgrass- and sagebrush-dominated soils warrant future research to examine soil fungal community composition across more sites and time points as

faculty startup funds from the Idaho State University Office of Research and the College of Science and Engineering. The funders had no role in study design, data collection and analysis, decision to publish, or preparation of the manuscript.

Competing Interests: The authors have declared that no competing interests exist.

well as in association with native grass species that also occupy cheatgrass-invaded ecosystems.

Introduction

Nonnative *Bromus tectorum* (cheatgrass) is rapidly invading and decimating sagebrush steppe (*Artemisia tridentata* and its congenerics), one of the most extensive ecosystem types in the Western U.S. [1]. This aggressive invasion has overtaken 22 million hectares and has been facilitated by livestock grazing and increased fire frequency that has decreased plant cover and increased nutrient availability in sagebrush steppe [2–6]. Loss of sagebrush equates to lost ecosystem services including service as nurse plants for seedlings and as habitat for unique assemblages of birds, mammals and insects [7–8]. Simultaneously, cheatgrass dramatically alters patterns of carbon (C) cycling and belowground C inputs in sagebrush steppe; as a winter annual, cheatgrass germinates in the fall or spring and grows actively in late spring, at the time most native perennials are just beginning to grow. In early summer, cheatgrass senesces and its aboveground biomass is typically completely dry by mid-July [9].

In addition to supporting macrofauna, sagebrush provides a habitat for unique assemblages of microorganisms in the form of biological crusts on the soil surface, a primary source of nitrogen (N) inputs in aridland ecosystems [10], and in sub-surface soils. Previous studies have demonstrated that cheatgrass invasion of aridland ecosystems can alter soil microbial community composition and structure as well as rates of microbially-mediated nutrient cycling processes [1,5, 11–15]. Specifically, shifts in soil microbial community composition in cheatgrass-invaded ecosystems have been shown using DNA fingerprinting methods (T-RFLP; [16]), while other studies have measured decreased fungal abundance, increased abundance of metabolically active bacteria, reduced species richness [17] and decreased abundance of arbuscular mycorrhizal fungi (AMF) associated with native plants [18–20].

Sagebrush hosts and is dependent on AMF for nutrient acquisition [21], while cheatgrass is a relatively poor AMF host and instead supports an abundance of dark septate endophytes (DSE) in its root system [22]. As a result, cheatgrass invasion shifts the predominant plant-fungal relationships in sagebrush steppe. Among other consequences, the limited ability of cheatgrass to host AMF may contribute to reduced C-sequestration. AMF enhance the formation and maintenance of soil macroaggregates in which C is protected, or sequestered, from degradation [23]. Macroaggregates are correlated with the presence of glomalin, a glycoprotein produced by AMF, and glomalin related soil proteins (GRSP) that turnover slowly in soil [24–25]. However, GRSP has not been quantified in studies of cheatgrass-invaded sagebrush steppe ecosystems, so the potential impacts on GRSP-related C-storage due to shifts from an AMF-sagebrush system to predominantly DSE-cheatgrass are unknown. In forested ecosystems, it has been shown that disruption of mycorrhizal fungi stimulates increases in opportunistic fungal abundances, and changes in overall fungal community composition [26]. Similar patterns may develop in sagebrush soils as a consequence of cheatgrass invasion.

It has been widely noted that belowground microbial composition plays a significant role in controlling invasive species spread, decomposition and C-sequestration in soils, but a mechanistic understanding of the linkages between changes in microbial composition and rates of ecological processes remains elusive [27–29]. While providing important insights, previous studies of cheatgrass invasion have lacked sufficient taxonomic resolution to determine which specific fungal taxa may be most susceptible to changes in relative abundance; in addition, they have not addressed vertical patterns in soil fungal community composition.

In the present study, we used high-throughput amplicon-sequencing of the ribosomal large subunit (LSU) gene to test the hypothesis that cheatgrass invasion may decrease vertical stratification of fungal community structure and composition in sagebrush steppe soils in the 0–4 cm and 4–8 cm depth intervals. Although neither fungal richness nor biomass varied statistically among the four soil fractions, the relative abundance of the fungal orders differed significantly between the sagebrush and cheatgrass-dominated soils. This first in-depth study of fungal taxonomic shifts in cheatgrass-invaded, sagebrush steppe soils, suggests that functional shifts towards saprotroph-dominated fungal communities may be occurring.

Materials and Methods

Sample collection

In September 2011, when cheatgrass was senesced, soil cores were collected from the Barton Road Ecological Research Area (Idaho State University, Pocatello, Idaho, USA; 42.853°N, 112.402°W), which has been previously described [30]. Six cores (7.5 cm diameter) were collected using aluminum core tubes from cheatgrass-invaded soils in a plot from which all sagebrush had been removed in 1996 ("shrub-removal plots" described by Inouye [31]). These six cores were collected randomly within the border (1 m X 20 m area) of one sagebrush removal plot (Fig. 1), which is dominated by cheatgrass (Inouye, personal communication; Fig. 1). At the time of sampling, cheatgrass had been impacting the study site for < 15 years, as shrubs, perennial grasses and forbs dominated the vegetation on these plots prior to shrub removal in 1996 [31]. Six additional cores were collected from underneath randomly chosen sagebrush shrubs in an unaltered area adjacent to the shrub-removal plot; these cores were all collected within 30 cm of the main trunk of a sagebrush shrub. Each core was sectioned into 0–4 cm and 4–8 cm depth intervals resulting in a total of 24 soil fractions. Soil fractions were designated as follows: "CT" (cheatgrass 0–4 cm), "CB" (cheatgrass 4–8 cm), "ST" (sagebrush 0–4 cm) and "SB" (sagebrush 4–8 cm). Immediately after collection, each fraction was manually homogenized in a clean ziptop bag. Two subsamples of the homogenized fractions (about 50 g each) were transferred to sterile, 50-mL disposable conical centrifuge tubes that were flash-frozen in liquid N_2; these samples were transported to the laboratory on dry ice where they were stored at -80°C until they were utilized for DNA extraction and glomalin-related soil protein (GRSP) analyses. The remainder of each of the fractions was stored in the ziptop bags under ambient conditions for transport; within 24 h, these samples were weighed and dried under ambient laboratory conditions in preparation for soil chemical and physical property analysis.

Soil properties and split plot analyses

Upon returning from the field, free water content was gravimetrically estimated for subsamples (140 g on average) from each soil fraction that were weighed and incubated under ambient room conditions (relative humidity: 25–30%, temperature: 25°C) until mass loss stabilized [32]. Soils were sieved (2 mm mesh) prior to additional analyses. Soil pH was measured using a Corning 430 pH meter after mixing soils with nanopure water (>17.5 MΩ) in a 1:1 ratio (w/v) and incubating them for 1 h under ambient conditions. For analyses of soil N and C content, samples were dried at 60°C overnight and then ground in a SamplePrep 8000M ball mill for 2 min. Ground soils (59 mg ± 1) were packed in silver capsules (5 mm x 9 mm) and acid fumigated to remove carbonates [33]. The capsules were closed and packed in tin capsules (5 mm x 9 mm). The %C and %N content in the soils were determined using the Costech ECS 4010 CHNSO Analyzer (Analytical Technologies, Inc., Valencia, CA) at the Interdisciplinary Lab for Elemental and Isotopic Analyses (Center for Archaeology, Materials and Applied Spectroscopy, Idaho State University, Pocatello, Idaho, USA).

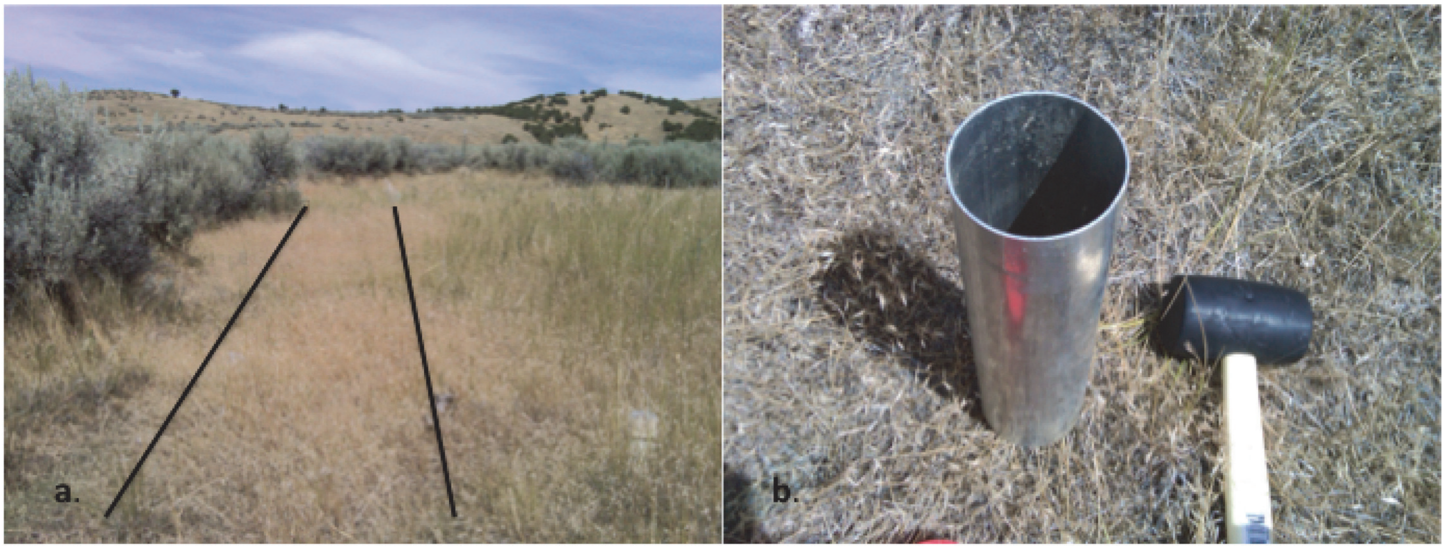

Figure 1. Shrub removal plot (a.) from which cheatgrass dominated soils (b.) were sampled and the adjacent area from which sagebrush-dominated soils were sampled. The black lines in panel a. outline the cheatgrass-dominated soils that were sampled in the 1 m × 20 m border of the plot; cheatgrass-dominated soils outlined in panel a. are shown up-close in panel b. Photos were taken by C.F. Weber at the time of sampling (September 2011).

doi:10.1371/journal.pone.0117026.g001

Glomalin-related soil proteins (GRSP) were quantified following the protocol of Bedini et al. [34]. Briefly, 1 g soil samples were transferred into 50 mL conical, disposable centrifuge tubes with 8 mL of 50 mM sodium citrate, pH 8.0. The soil solutions were autoclaved for 60 min and centrifuged (5000 x g, 20 min) to pellet the soil. The supernatants from each sample were decanted, and then pooled with the supernatant from a second identical extraction of the same sample. In a preliminary analysis, two rounds were sufficient to extract > 90% of total GRSP, which was analyzed using the Bradford Assay according to manufacturer's protocol for using the Bradford Reagent (Brilliant Blue G, Sigma-Aldrich, Inc. [35]). Briefly, extracts to be analyzed were mixed with the Bradford Reagent in a ratio of 1 part sample to 30 parts Bradford Reagent (3.1 mL final volume) and the absorbance of the resulting protein-dye complex was measured using a spectrophotometer set to a wavelength of 595 nm. The assay was calibrated with bovine serum albumin (BSA) standards.

As a conventional response to their strictly bounded units, data for %C, %N and %water content were arcsine square-root transformed. Data for all soil properties listed in Table 1 were then examined for normality using Shapiro Tests and homoscedasticity using the Fligner-Killeen Tests. Data for soil properties that had P-values > 0.05 for diagnostic tests of normality and homoscedasticity were analyzed using a split-plot ANOVA with vegetation type as the whole plot factor and depth as the split plot factor. Thus, these models had the form:

$$Y_{ijk} = \mu + \text{vegetation type}_i + \text{site}_{j(i)} + \text{depth}_k + (\text{vegetation type} \times \text{depth})_{ik} + \text{Error}_{ijk}$$

where μ is a constant that varies with the specified linear model contrast, Y_{ijk} represents the mean response for the ith vegetation type at the kth depth and the jth site (in the ith vegetation type) [36].

Statistical results (F-statistic; d.f.; P-value) are reported only where P-values were ≤ 0.05. There was no significant effect of the interaction between veg. type and depth on any of the soil properties. For cases in which the P-value > 0.05, "nse" (no significant effect) is reported. CT = cheatgrass surface soil (0–4 cm), CB = cheatgrass subsurface soil (4–8 cm), ST = sagebrush

Table 1. Average (n = 6 (±1 standard error)) physical, chemical and biological properties of soil intervals and the results of a split-plot ANOVA.

Soil Fraction	%N	%C	C:N ratio	pH	Water Content (%)	GRSP mg(g soil)$^{-1}$	Fungal 18S rRNA Gene Copy No.
CT	0.16 (0.01)	1.94 (0.19)	12.0 (0.54)	7.30 (0.12)	2.57 (0.56)	3.95 (0.34)	$5.45x10^{7}$ ($4.99x10^{7}$)
CB	0.10 (0.003)	0.96 (0.03)	9.5 (0.19)	7.91 (0.09)	5.73 (0.29)	2.81 (0.34)	$3.79x10^{8}$ ($3.57x10^{8}$)
ST	0.20 (0.02)	3.05 (0.28)	14.9 (0.40)	7.44 (0.17)	1.37 (0.40)	5.75 (0.66)	$7.23x10^{6}$ ($1.59x10^{6}$)
SB	0.11 (0.003)	1.52 (0.10)	13.4 (0.86)	8.22 (0.02)	3.98 (0.17)	2.53 (0.12)	$6.06x10^{7}$ ($1.97x10^{7}$)
Split-plot ANOVA							
veg. type	6.37; 1,9.94; 0.030	22.26; 1,22; 0.0008	28.41; 1,22; 0.0003	nse	15.71; 1,8.63; 0.0036	nse	nse
depth[1]	190.6; 1,∞; 0	48.11; 1,22; 4.0 $x10^{-5}$	19.68; 1,22; 0.0013	36.7; 1,22; 0.00012	103.9; 1,∞; 0	21.7; 1,∞; 3.2 $X10^{-6}$	nse

[1]As noted in the results section, in the method of Brunner et al. [37], the denominator degrees of freedom for the test for split plot effects and the interaction of whole and split plots will be infinite. As a practical matter, the upper tailed probabilities for large and infinite denominator degrees of freedom will be subequal. Thus, the code for this algorithm in [38] uses 10,000 denominator degrees of freedom for these tests.

doi:10.1371/journal.pone.0117026.t001

surface soil (0–4 cm), SB = sagebrush subsurface soil (4–8 cm); GRSP = glomalin-related soil proteins.

For data that had *P*-values $\leq$ 0.05 for diagnostic tests, a nonparametric, rank-based approach to split-plot design robust to heteroscedasticity described by Brunner et al. [37] was used to test for significant effects of vegetation type, depth and the interaction between the two using R-code written by Wilcox [38]. The R computational environment [39] was used for all parametric and nonparametric split plot analyses.

Soil DNA extraction, PCR and sequencing

Total DNA was extracted from one 0.25–0.50 g subsample of each of the 24 flash-frozen soil fractions using a MoBio Powersoil DNA extraction kit (MoBio Laboratories, Carlsbad, California, USA). Extractions were performed as described by the manufacturer, with the exception of using 50 μL volumes to elute purified DNA from the spin columns in the final step of the protocol. Ribosomal LSU gene fragments were amplified in triplicate from each DNA extract for sequencing on the Roche 454 GS-FLX Titanium Platform using barcoded primers as previously described in Weber et al. [40]. All reactions included the primer 454_LROR, consisting of the GS-FLX 'B' adapter sequence fused to the 5' end of the LR0R primer sequence and a unique version of the primer 454_LR3 in which a five-base identifying barcode was flanked by the GS-FLX 'A' adapter sequence on the 5' end, and the LR3 primer sequence on the 3' end. Each 25 μL reaction contained 1.5 units of AmpliTaq DNA polymerase (Applied Biosystems, Carlsbad, California, USA), 1X AmpliTaq Buffer II, 800 μM dNTPs, 0.4 μM each primer, 1.5 mM $MgCl_2$ and 6 μg BSA. Samples were initially denatured at 95° C for 3 min followed by 30 cycles of 95° C for 1 min, 55° C for 1 min and 72° C for 1 min and a final 10 min extension at 72° C. Successful amplification in each reaction was confirmed by resolving products on a 1% TAE gel and staining with ethidium bromide. Triplicate reactions were pooled before purification and concentration using the Qiagen MinElute PCR Cleanup kit (Qiagen, Valencia, California, USA). An optional 35% guanidine-HCl wash step was performed to ensure that all large primer-dimers were removed from the samples. For sequencing, equimolar quantities of amplicons were pooled and submitted to the Duke University Genome Sequencing & Analysis Core Resource (Durham, North Carolina, USA).

Quantitative PCR of the fungal 18S rRNA gene

Relative abundance of fungal 18S rRNA gene copy numbers was examined in all 24-soil fractions from the same DNA extracts that were utilized for the sequence libraries. Quantitative PCR was carried out using primers described by Castro et al. [41] as detailed in Weber et al. [40] in an MJ Research DNA Engine DYAD. Gene copy number was quantified three times for each of the six replicate soil samples collected per soil interval. After log transformation, the Shapiro and Fligner Killeen diagnostic tests for this data had *P*-values of > 0.05 and thus were statistically examined for the effects of vegetation, depth and an interaction of the two using conventional split-plot ANOVAs as described above.

Sequence data processing and analysis

Sequence data were analyzed using the mothur software package [42]. Sequences were parsed based on the presence of the LR3 primer sequence preceded by the unique barcode sequence. Sequences that did not contain exact matches to the primer and barcodes utilized in the PCR amplification were discarded. Following identification, sequences were filtered for quality with a 50-base sliding window with a minimum average quality score of 25. Additionally, sequences containing ambiguous bases, homopolymers $>$ seven bases and with lengths $<$ 475 bp were eliminated from the dataset. All sequences longer than 475 bp were trimmed to this length. Sequences for each sample were then aligned to a reference alignment consisting of fungal LSU sequences from the AFTOL database (http://aftol.org/). Any sequences that could not be aligned, or that produced alignments too short or too long, were discarded. Chimeras were identified in the aligned sequences using the pintail algorithm and discarded. Sequences were clustered into OTUs using the average neighbor algorithm and a similarity cutoff of 97%. Rarefaction curves, diversity indices (i.e., Ace, Chao1, inverse Simpson and Shannon), richness and rank abundance curves were generated after randomly subsampling 7,000 sequences from each of the 24-libraries to normalize the calculations. Using the otu.rep command in mothur, representative sequences for each OTU were selected and were classified taxonomically using the Ribosomal Database Project's (RDP) online classifier for fungal LSU genes [43]. Taxonomic composition of each library was determined based on the classification of sequences in the RDP. Any sequences that did not classify within the fungal domain at 100% confidence were eliminated from taxonomic analyses. Sequences were only assigned to a particular taxon if they classified with $\geq$80% confidence. Composition data of each library (proportions) was utilized to calculate distance matrices and complete multidimensional scaling analysis (MDS) in the vegan package of R [39] based on the BrayCurtis dissimilarity metric [44] at the order level. Multivariate regressions were used to quantify the capacity of edaphic properties to explain variance in the summarized community space of the ordination. *P*-values for regression predictors were obtained from the function envfit in the vegan package in R [39, 45] using 1000 permutations. To determine if the relative proportion of fungal taxa (arcsine square root transformed), normalized richness, or diversity indices were significantly affected by vegetation type, depth or an interaction of the two factors, data were examined for normality and homoscedasticity and then subjected to parametric or nonparametric split-plot ANOVAs as appropriate, as described above for soil properties.

Sequence accession

Sequences have been deposited into MG-RAST (http://metagenomics.anl.gov) under the following ID numbers: 4574367.3, 4574369.3, 4574371.3, 4574373.3, 4574375.3, 4574377.3, 4574379.3, 4574381.3, 4574383.3, 4574385.3, 4574387.3, 4574389.3, 4574366.3, 4574368.3,

4574370.3, 4574372.3, 4574374.3, 4574376.3, 4574378.3, 4574380.3, 4574382.3, 4574384.3, 4574386.3, 4574388.3.

Results

Soil properties

Measurements of soil chemical and physical properties for surface (0–4 cm) and subsurface (4–8 cm) fractions of sagebrush and cheatgrass soils are summarized in Table 1. Vegetation type had a significant impact on %N (F = 6.37, df = 1, 9.17, P-value = 0.030), % C (F = 22.26, df = 1, 9.22, P-value = 0.0008), C:N ratio (F = 28.41, df = 1, 22, P-value = 0.0003) and water content (F = 15.71, df = 1, 8.63, P-value = 0.0036). We note that %N, %C and C:N ratio were greater in sagebrush soils than in cheatgrass soils, but the opposite was true for water content (Table 1). Note that the nonparametric split plot analyses denominator degrees of freedom for F-statistics were non-integers due to Satterthwaite adjustment for heteroscedasticity. Depth had a significant effect on all six soil properties (all P-values $\leq$ 0.0013, Table 1). Average free water contents were between 2.2 and 2.9 times higher in the subsurface than in the surface soils for both vegetation types; likewise, pH was between 0.6 and 0.8 units higher in the subsurface than in the surface soils in both vegetation types. In contrast, total nitrogen (%N) and organic carbon (%C) contents were 1.6 to 1.8 and 2 times greater, respectively, in the surface than in the subsurface intervals in both vegetation types. Average GRSP content was 1.4 to 2.2 times higher in the surface than in the subsurface layer. The interaction between vegetation type and depth did not significantly impact any of the six soil properties (all P-values $>$ 0.16, Table 1).

Fungal richness, diversity and community structure

Across all 24-soil fractions, a total of 333,047 sequences passed the quality control criteria outlined in the materials and methods section. The average library size was 13,877 sequences with libraries ranging in size from 7,037 to 21,002 (S1 Table). Normalized OTU-based richness, diversity indices and evenness are in Table 2. The average OTU-based richness of sequence libraries was not significantly affected by vegetation (F = 3.38, df = 1, 22, P-value = 0.096), depth (F = 0.46, df = 1, 22, P-value = 0.51),) or their interaction (F = 0.0006, df = 1, 22, P-value = 0.98); however, normalized richness calculations and rarefaction curves indicated that the ST and SB sequence libraries (ST = 1,341 ± 160; SB = 1,250 ± 51) tended to harbor greater richness than CT and CB sequence libraries (CT = 1,125 ± 134; CB = 1,041 ± 116), respectively, and within each vegetation type, the 0–4 cm soil interval tended to harbor greater richness than the 4–8 cm intervals (Table 2; S1 Fig.). The inverse Simpson diversity index (ST = 9.9 ± 7.3, SB = 29.1 ± 6.8, CT = 12.8 ± 4.5, CB = 13.2 ± 5.8) was significantly impacted by vegetation type (F = 6.50, df = 1, 22, P-value = 0.029); Ace, Chao1, and Shannon indices were not significantly affected by vegetation type (all F $<$ 2.36, df = 1,22, all P-values $>$0.16), depth (all F $<$3.12, df = 1, 22, all P-values $>$0.11), or the interaction between the two factors (all F $<$1.01, df = 1, 22, all P-values $>$0.34). However, the average Chao1 (ST = 5408 ± 835, SB = 4050 ± 498, CT = 4341 ± 582, CB = 3969 ± 355) and Ace (ST = 11,896 ± 2192, SB = 9535 ± 561, CT = 8848 ± 1321, CB = 8008 ± 684) indices tended to be slightly higher in ST intervals than the others, while the average Shannon index (ST = 4.7 ± 0.5, SB = 5.0 ± 0.2, CT = 4.4 ± 0.3, CB = 4.4 ± 0.3) tended to be higher in the SB intervals than in the other three soil intervals. Diversity differed between depth intervals in sagebrush soils more than in cheatgrass soils; for every diversity index, CT and CB had very similar average values, which was not the case for ST and SB (Table 2). Although not statistically significant, Simpson evenness was highest in SB fractions and was evident in the rank abundance curves (S2 Fig.).

Table 2. Average normalized richness (7,000 sequences per sequence library) and diversity indices for each of the four soil intervals (n = 6 (±1 standard error)).

Soil Fraction	Chao1	Ace	Shannon	Inverse Simpson*	Simpson Evenness	OTUs
CT	4340 (582)	8848 (1321)	4.43 (0.28)	12.8 (4.5)	0.016 (0.002)	1125 (134)
CB	3969 (355)	8008 (684)	4.43 (0.35)	13.2 (5.8)	0.023 (0.004)	1041 (116)
ST	5408 (835)	11896 (2192)	4.74 0.52)	9.9 (7.3)	0.025 (0.004)	1341 (160)
SB	4050 (498)	9535 (561)	4.96 (0.20)	29.1 (6.8)	0.032 (0.005)	1250 (51)

OTU's were defined at a maximum distance of 0.03.

"*" denotes statistically significant effect of vegetation type (F = 6.50, d.f. = 1,22, P-value = 0.029).

None of the richness or diversity indices were significantly affected by soil depth or the interaction between vegetation type and soil depth (all P-values > 0.05). CT = cheatgrass surface soil (0–4 cm), CB = cheatgrass subsurface soil (4–8 cm), ST = sagebrush surface soil (0–4 cm), SB = sagebrush subsurface soil (4–8 cm).

doi:10.1371/journal.pone.0117026.t002

Fungal community composition

Of all sequences that passed the quality control criteria, 92% (307,936) classified within the fungal domain with 100% confidence (Ribosomal Database Project Fungal classifier) and were utilized in subsequent compositional analyses (S1 Table). Of these sequences, 99%, 95% and 88% classified at the phylum, class and order levels, respectively, with $\geq$ 80% confidence (S1 Table).

At the phylum level, Ascomycota comprised the largest percentage of sequences recovered from all soil fractions (Fig. 2A); distribution of Ascomycota among the sequence libraries was significantly impacted by depth (F = 6.16, df = 1, ∞, P-value = 0.013) with greater abundance noted in the CT (83.8% ± 9.8%) than in the CB (66.3% ± 7.9%), ST (67.3% ± 11.3%) and SB (64.6% ± 4.0%) libraries (Table 3). Note that in the method of Brunner et al. [37] the F-statistic for both split plot effects main effects, and for the interaction of whole and split plots, will have in infinite denominator degrees of freedom under H_0. Thus, the denominator degrees of freedom in these tests will be unaffected by sample size. Although it was not statistically significant, the opposite trend was observed for Basidiomycota (Fig. 2A), with lower values for CT (16.1% ± 9.8%) than for CB (31.9% ± 8.0%), ST (32.4% ± 11.3%) and SB (26.7% ± 3.4%). Sequences classified as Blastocladiomycota, Chytridiomycota, Fungi incertae sedis and Glomeromycota comprised, on average, less than 1% of the sequences, with the exception of the SB libraries, for which Fungi incertae sedis and Chytridiomycota comprised, on average, about 3% and nearly 5% of sequences, respectively (Fig. 2B). The distribution of Glomeromycota among the sequence libraries was significantly impacted by depth (F = 12.05, df = 1, ∞, P-value = 0.0005; Table 3) with greater abundances found in the 4–8 cm intervals in both vegetation types (Fig. 2; Table 3). The distribution of Fungi incertae sedis and Chytridiomycota were significantly impacted by depth and vegetation type (all P-values < 0.024, Table 3) with the greatest abundance being detected in the SB fractions for both taxon classifications. The distribution of Chytridiomycota was also impacted by the interaction of vegetation type and depth (F = 6.44, df = 1,∞, P-value = 0.011).

Numbers in each row indicate the F statistic, d.f. and P-value. For cases in which P-values > 0.05, "nse" (no significant effect) is reported.

While MDS plots created based on the phylum-level classification of OTU's did not reveal distinct clustering of libraries by vegetation type or depth, MDS plots constructed based on order-level classification of OTU's revealed, with the exception of library S7B, all SB sequence libraries cluster more closely together than the ST, CT and CB libraries (Fig. 3). Cheatgrass sequence libraries, in general were scattered throughout the plot indicating that much greater

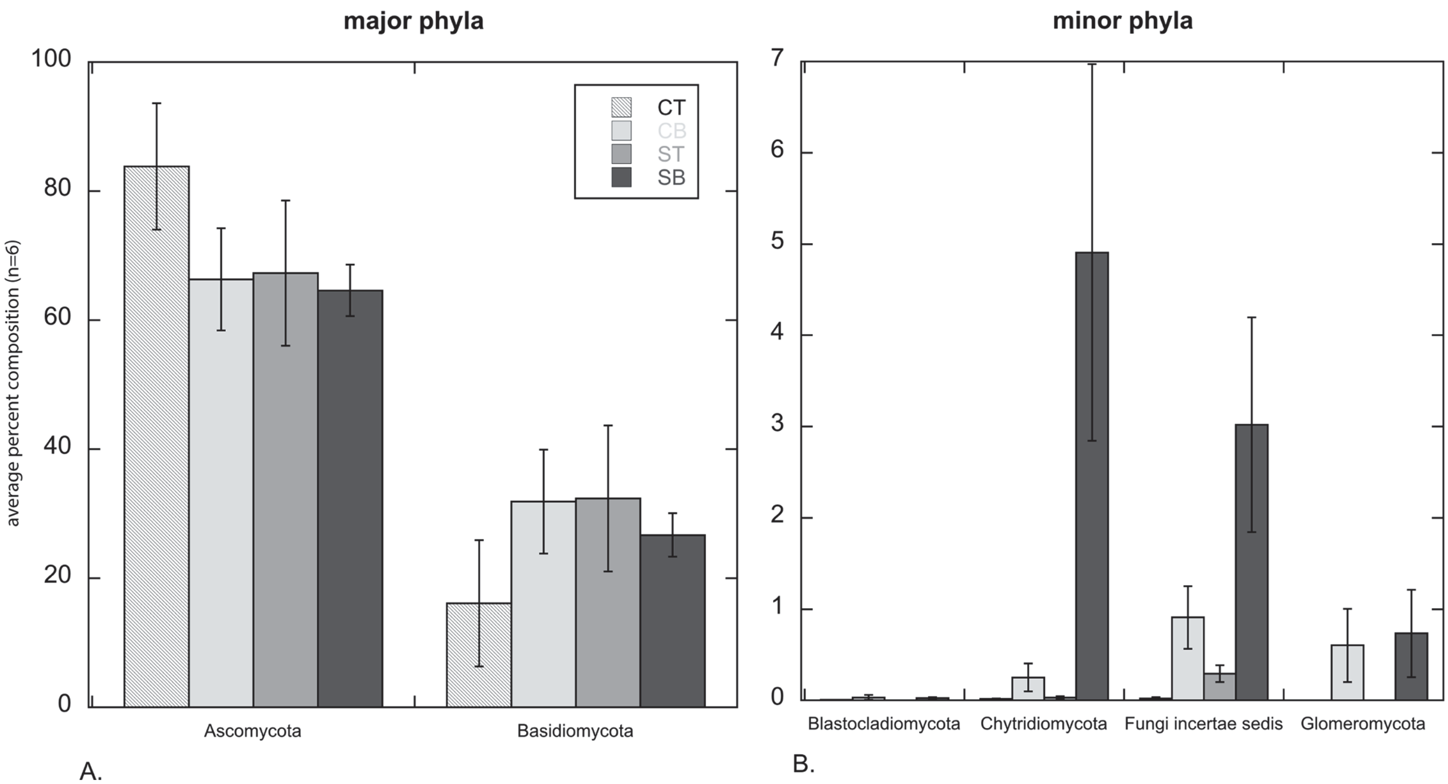

Figure 2. The average percentage of sequences for each soil interval that classified within a particular fungal phylum ($n = 6 \pm 1$ standard error). Soil intervals are designated as CT (cheatgrass 0–4 cm), CB (cheatgrass 4–8 cm), ST (sagebrush 0–4 cm) and SB (sagebrush 4–8 cm). Where bars are absent, the phylum was absent from all replicates.

doi:10.1371/journal.pone.0117026.g002

Table 3. Fungal phyla and orders for which vegetation type, depth and/or an interaction between the two significantly impacted their proportion in the sequence libraries.

Phylum	Vegetation Type	Depth[1]	Vegetation Type x Depth[1]
Ascomycota	nse	6.16; 1,∞; 0.013	nse
Chytridiomycota	12.59; 1,7.11; 0.0091	7.22; 1,∞; 0.0072	6.44; 1,∞; 0.011
Fungi incertae sedis	7.22; 1,9.62; 0.024	19.81; 1,∞; 8.56 x10–6	nse
Glomeromycota	nse	12.05; 1,∞; 0.0005	nse
Order			
Cantharellales	29.34; 1,9.28; 0.00038	nse	nse
Coniochaetales	5.54; 1,7.86; 0.047	14.21;1,∞; 0.00016	nse
Pezizales	12.38; 1,6.24; 0.012	27.11; 1,∞; 1.93 x 10^{-7}	nse
Spizellomycetales	8.03; 1,7.98; 0.022	18.56; 1,∞; 1.65 x10^{-5}	7.47; 1,∞; 0.0063
Teloschistales	12.16; 1,9.27; 0.0065	25.38; 1,∞; 4.71 x 10^{-7}	7.61; 1,∞; 0.0058
Capnodiales	nse	68.04 1,∞; 2.22x10^{-16}	11.06; 1,∞; 0.0009
Sordariales	nse	14.03; 1,∞; 0.0002	11.44; 1,∞; 0.0007

[1]As noted in the results section, in the method of Brunner et al. [37], the denominator degrees of freedom for the test for split plot effects and the interaction of whole and split plots will be infinite. As a practical matter, the upper tailed probabilities for large and infinite denominator degrees of freedom will be subequal. Thus, the code for this algorithm in [38] uses 10,000 denominator degrees of freedom for these tests.

doi:10.1371/journal.pone.0117026.t003

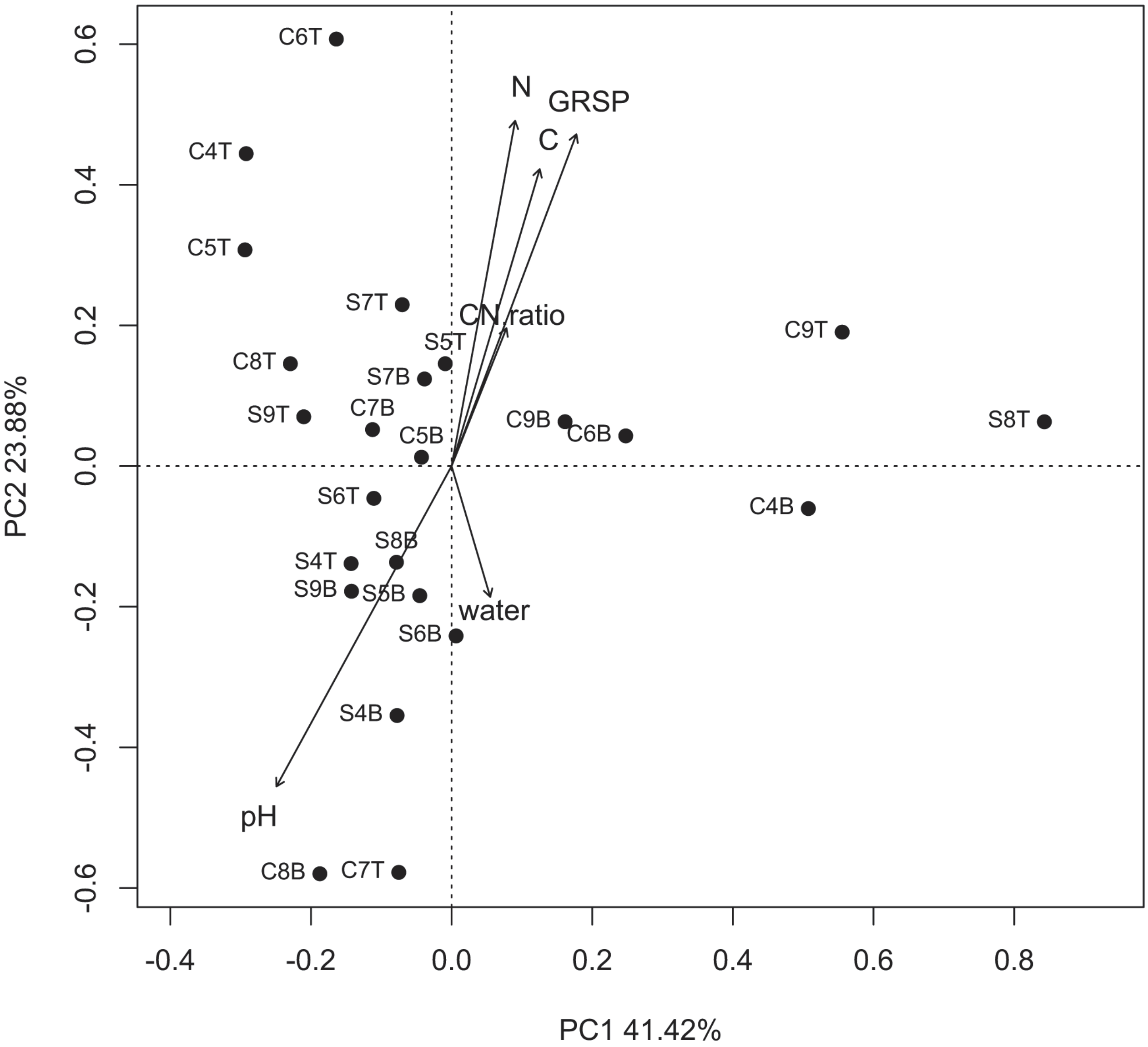

Figure 3. Multidimensional scaling plot (Bray-Curtis dissimilarity metric) displaying the relatedness of sequence library composition based on order-level classification. Soil intervals and sequence libraries from them are designated as CT (cheatgrass 0–4 cm), CB (cheatgrass 4–8 cm), ST (sagebrush 0–4 cm) and SB (sagebrush 4–8 cm). Each sequence library is accompanied with a sample number (4, 5, 6, 7, 8 or 9). Arrows represent projections of soil variables (water content (water), glomalin related soil protein (GRSP), % carbon (C), % nitrogen (N), C:N ratio and pH) relative to community composition.

doi:10.1371/journal.pone.0117026.g003

compositional heterogeneity at the order-level was present among cheatgrass sequence libraries than among sagebrush sequence libraries (Fig. 3). At the order level, the first principal component accounted for 41.42% of the variability, while the second dimension accounted for 23.88% of the variability, coarsely separating 0–4 cm from 4–8 cm sequence libraries in both vegetation types. GRSP (R^2 = 0.1545 *P*-value = 0.171), %N (R^2 = 0.1516 *P*-value = 0.178), %C (R^2 = 0.1180 *P*-value = 0.271), C:N ratio (R^2 = 0.0271, *P*-value = 0.750), increased in the positive direction along the second dimension, while pH (R^2 = 0.1639, *P*-value = 0.161) and water content (R^2 = 0.0229 *P*-value = 0.784) increased in the negative direction along the second dimension with pH having a skew towards the primary cluster of SB libraries.

Table 4. Average percentage of the 19 most abundant fungal orders detected in each of the four soil intervals (n = 6 (±1 standard error)).

Order	CT	CB	ST	SB
Pleosporales	40.48 (10.53)	18.91 (4.04)	26.55 (5.08)	19.40 (3.06)
Agaricales	14.19 (10.44)	24.19 (8.70)	21.81 (12.95)	9.79 (1.80)
Helotiales	9.09 (2.64)	16.27 (9.83)	11.66 (2.98)	11.31 (2.45)
Pezizales	2.77 (1.77)	9.45 (2.13)	5.18 (2.86)	24.79 (4.80)
Cantharellales	1.30 (0.67)	1.39 (0.93)	6.52 (2.12)	12.24 (2.24)
Sordariales	3.26 (1.16)	11.54 (3.20)	2.47 (0.94)	2.39 (0.48)
Coniochaetales	7.09 (2.45)	3.97 (3.22)	3.00 (1.29)	0.20 (0.10)
Hypocreales	5.17 (2.08)	4.66 (1.92)	2.39 (0.70)	1.79 (0.78)
Polyporales	9.85 (9.83)	0.02 (0.02)	0.31 (0.29)	0.56 (0.55)
Chaetothyriales	0.84 (0.22)	0.77 (0.41)	4.63 (2.00)	0.56 (0.25)
Spizellomycetales	0.01 (0.01)	0.28 (0.17)	0.001 (0.003)	5.15 (2.30)
Capnodiales	1.63 (0.69)	0.56 (0.25)	2.74 (0.89)	0.07 (0.03)
Tremellales	0.15 (0.04)	0.13 (0.03)	1.17 (0.24)	2.89 (2.27)
Eurotiales	0.25 (0.08)	0.86 (0.23)	1.59 (0.78)	1.12 (0.28)
Xylariales	1.06 (0.45)	0.53 (0.11)	0.96 (0.71)	0.40 (0.14)
Auriculariales	0.11 (0.10)	1.36 (1.36)	0.04 (0.03)	1.38 (1.02)
Microascales	0.07 (0.02)	2.27 (1.99)	0.02 (0.01)	0.42 (0.16)
Teloschistales	0.006 (0.004)	-	1.28 (0.64)	0.01 (0.01)
Acarosporales	-	-	1.02 (1.02)	-

All orders listed comprised 1% or more, on average, of the sequences from one or more of the four soil intervals. CT = cheatgrass surface soil (0–4 cm), CB = cheatgrass subsurface soil (4–8 cm), ST = sagebrush surface soil (0–4 cm), SB = sagebrush subsurface soil (4–8 cm). "-" indicates none detected.

doi:10.1371/journal.pone.0117026.t004

On average, 19 fungal orders each comprised ≥ 1% of sequences recovered from one or more of the four soil intervals (Table 4). On average, Pleosporales was most abundant in CT (40.5%) and ST (26.5%) libraries, while Agaricales was most abundant in CB libraries (24.2%) and Pezizales was most abundant in SB libraries (24.8%). Among the 19 orders, the relative abundance of seven orders were significantly impacted by vegetation type, depth or the interaction between the two factors (Fig. 4; Table 3). Cantharellales was significantly impacted by vegetation type only (F = 29.34, df = 1, 9.28, P-value = 0.00038) with average abundance being higher in ST and SB fractions than in CT and CB fractions, respectively (Fig. 4). The distribution of the remaining six orders (Coniochaetales, Pezizales, Spizellomycetales, Teloschistales, Capnodiales, Sordariales) were all significantly impacted by depth (all P-values $\leq$ 0.0002, Table 3); four of these orders were also significantly impacted by the interaction between vegetation type and depth (Spizellomycetales, Teloschistales, Capnodiales, Sordariales) and four orders (Coniochaetales, Pezizales, Spizellomycetales, Teloschistales) were also significantly impacted by vegetation type (all P-values $\leq$ 0.047, Table 3). On average, Coniochaetales was most abundant in CT fractions, but Spizellomycetales and Pezizales were most abundant in SB and Teloschistales was most abundant in ST fractions (Fig. 4).

Several orders made up only small percentages of sequence libraries (an average of 0.001 to 1% in a given library); these orders contributed to the compositional uniqueness of the four soil intervals studied. Five orders (Acarosporales, Arthoniales, Botryosphaeriales, Candelariales, Microthyriales) were only found in ST, four orders (Atractiellales, Boletales, Monoblepharidales, Sebacinales) were only found in SB, and three orders (Pyxidiophorales, Phyllachorales, Agaricostilbales) were found in both sagebrush fractions but not in cheatgrass fractions. In contrast, relatively few orders were found in cheatgrass only: Cystofilobasidiales and Taphrinales

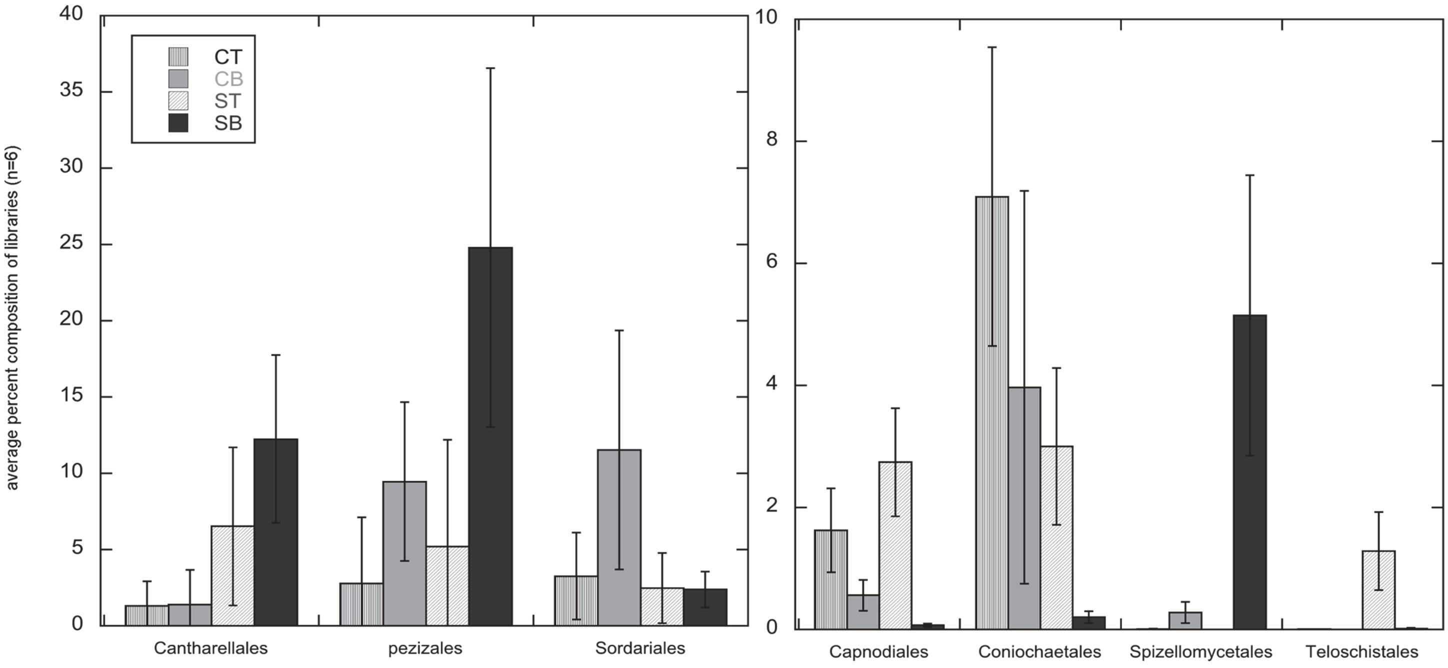

Figure 4. Average percentage of sequences (n = 6 (±1 standard error)) from each soil fraction type that classified into seven of the most abundant orders for which abundance was significantly impacted by vegetation type, depth or their interaction (*P*-values $<$ 0.05; Table 3). Soil intervals and sequence libraries from them are designated as CT (cheatgrass 0–4 cm), CB (cheatgrass 4–8 cm), ST (sagebrush 0–4 cm) and SB (sagebrush 4–8 cm). Note the differences in the scale of the y-axes in the two panels. Teloschistales was not detected in the CB fractions; other fractions for which there is no visible data had percentages less than 0.01.

doi:10.1371/journal.pone.0117026.g004

occurred exclusively in CT, while Russulales, Diaporthales and Magnaporthales were detected only in CB fractions.

Among the 19 most abundant orders, four comprised, on average, $\geq$ 1% of the sequences in each of the four soil intervals: Pleosporales, Agaricales, Helotiales and Hypocreales. The distribution of these orders across the sequence libraries was not significantly impacted by vegetation type, depth or the interaction between the two factors (Table 3), but the genus level composition within the orders did vary. The genus level composition within each of these four orders was examined in each of the four soil intervals by pooling the six replicate sequence libraries for each soil interval into "composite libraries" (Table 5). Within the Hypocreales, the recovery rate of genera was as much as 29 times higher in the SB composite library relative to the cheatgrass composite libraries (Fig. 5), even though the percentage of sequences that classified at the genus level in SB composite libraries was the lowest of the four composite libraries (Table 5). Within the Pleosporales, the numbers of genera observed were similar for each of the composite libraries, but the percentages of sequences that could be classified in each composite library were relatively low (27.1 to 43.2%) in comparison to the other three orders (Table 5). On average, 66% of the Agaricales, 60% of the Helotiales and 68% of the Hypocreales sequences were classified at the genus level with $\geq$ 80% confidence.

Composite library sequences that classified at the genus level within the Agaricales and Pleosporales were dominated by one or two genera. Agaricales sequences were dominated by *Gastrocybe* (84.0%) in CB, *Psathyrella* (61.0%) in SB, *Lepiota* (89.6%) in CT and *Clitopilus* (95.5%) in ST libraries (S2 Table). *Phaeosphaeria* dominated Pleosporales sequences from CT (71.9%), while more than one genus comprised the bulk of the Pleosporales sequences from each of the other soil fractions: *Phaeosphaeria* (39.7%) and *Alternaria* (33.1%) in ST;

Table 5. The number of and percentage of sequences classified at the genus-level (with ≥80% confidence) within the four most abundant fungal orders detected in the composite libraries, as well as the number of genera they include.

	CT	ST	CB	SB	Total
Agaricales					
No. of sequences	15,647	18,710	17,141	5,947	57,445
% sequences classified	91.4	72.8	44.5	41.7	
genera	17	16	14	40	87
Helotiales					
No. of sequences.	6,803	6,929	12,463	7,668	33,863
% sequences classified	45.9	61.4	88.3	23.9	
genera	4	7	5	20	36
Hypocreales					
No. of sequences	4,060	1,219	2,371	1,197	8,847
% sequences classified	95.3	59.6	45.6	32.0	
genera	10	11	9	29	59
Pleosporales					
No. of sequences	40,030	14,237	9,669	12,736	76,672
% sequences classified	43.2	27.1	28.6	38.4	
genera	27	23	21	32	103

Composite libraries for each of the four soil intervals include all six replicate libraries. CT = cheatgrass surface soil (0–4 cm), CB = cheatgrass subsurface soil (4–8 cm), ST = sagebrush surface soil (0–4 cm), SB = sagebrush subsurface soil (4–8 cm).

doi:10.1371/journal.pone.0117026.t005

Pyrenochaeta (33.0%) and *Phaeospharia* (25.9%) in CB; *Montagnula* (44.9%) and *Lophiostoma* (26.6%) in SB libraries (S3 Table).

The numbers of genera detected among sequences classified within the Hypocreales and Helotiales were highest in the SB soils (Table 5; Fig. 5). Within the Hypocreales, the most frequently recovered genus comprised only 26% of the classified sequences in SB (*Penicillium*; S4 Table), with the remainder of the sequences distributed among 28 other genera. In contrast, the Hypocreales sequences within the CT libraries were overwhelmingly dominated by *Gibberella* (98%), while the ST sequences were dominated by *Gibberella* (36.9%) and *Hypocrea* (33.4%) and the CB sequences were dominated by *Gibberella* (52.2%) and *Hydropisphaera* (38.7%) (S4 Table); the remainder of the sequences in the ST, CT and CB soil fractions were distributed among only nine, nine and seven genera, respectively. Similarly, the classified sequences within the Helotiales were distributed among three to five times more genera in the SB fraction than in any of the other three soil fractions (Table 5). The most frequently recovered genera among sequences within the Helotiales were *Sclerotinia* (46.9%) and *Cudoniella* (43%) in CT, *Tetracladium* (79%) in ST, *Cudoniella* (87%) in CB and *Tetracladium* (61.3%) in SB (S5 Table).

Discussion

As the first in-depth sequencing analysis of soil fungal communities in cheatgrass-invaded sagebrush steppe, this study provides a foundation upon which hypotheses can be built regarding mechanisms driving fungal community shifts in this ecosystem. The text that follows discusses the results of this study in the context of such hypotheses, while fully recognizing that this study's limitations prohibit conclusions from being drawn regarding the primary drivers of fungal community shifts and extrapolating them to large spatial scales (i.e. landscape, regional). These limitations include 1) studying fungal community structure and composition at

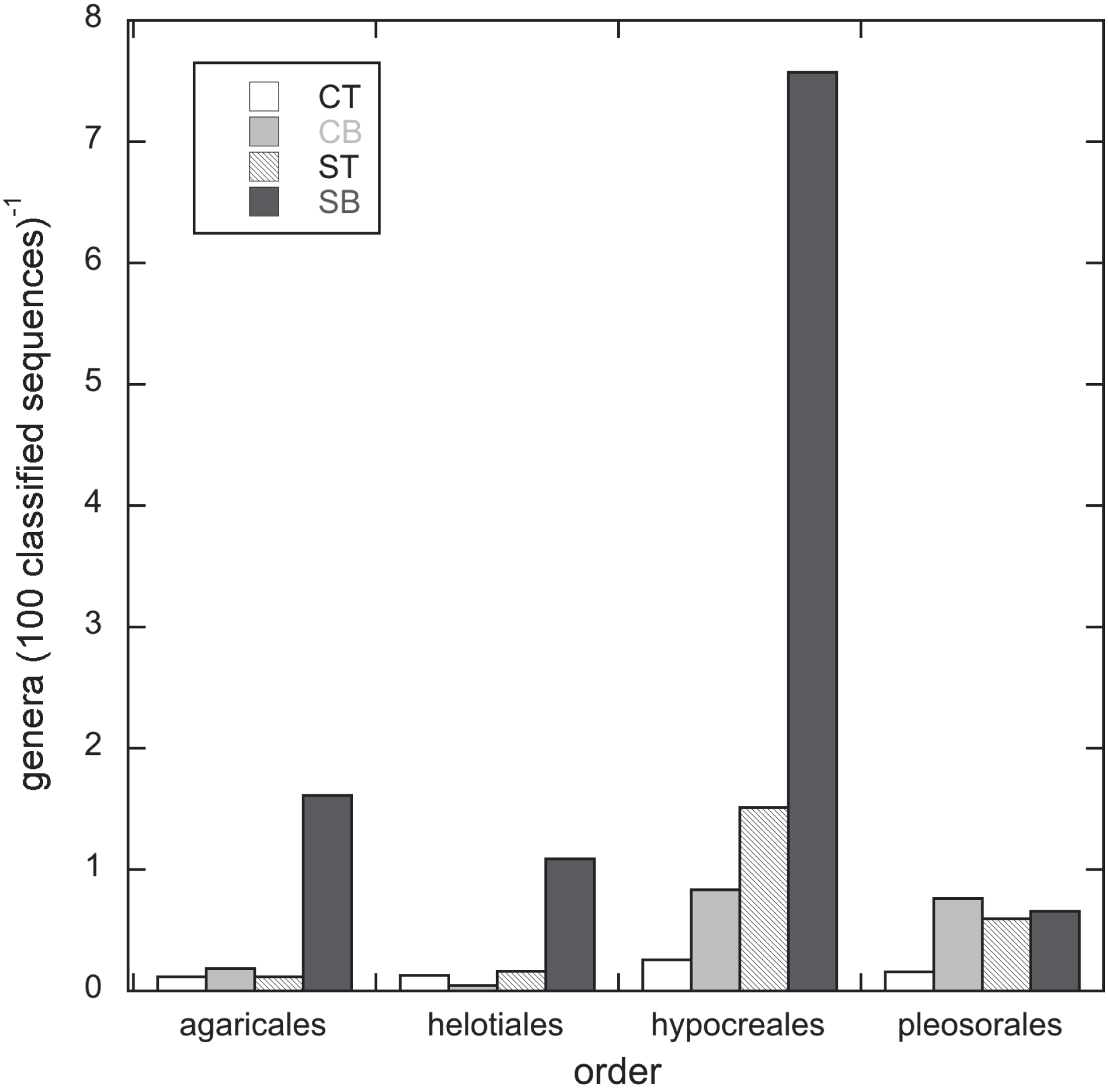

Figure 5. The number of genera recovered (100 sequences classified)$^{-1}$ in composite libraries for CT, ST, CB and SB libraries. Composite libraries for each of the four soil intervals include the libraries generated from the six field replicates. Sequences represented were classified at the genus level with $\geq$ 80% confidence in the Ribosomal Database Project Classifier. Soil intervals and sequence libraries from them are designated as CT (cheatgrass 0–4 cm), CB (cheatgrass 4–8 cm), ST (sagebrush 0–4 cm) and SB (sagebrush 4–8 cm).

doi:10.1371/journal.pone.0117026.g005

a single time point at one study site and 2) sampling cheatgrass-dominated soils from a "sagebrush removal plot" [31]. Additionally, we cannot be certain whether cheatgrass-invasion resulted in the fungal communities and soil properties that we observed or if the latter facilitated cheatgrass invasion. Recent studies suggest that fluctuations in resource availability (e.g. water) may facilitate cheatgrass invasion [46] and the resilience of spatial patterning of resources beneath shrubs and in shrub interspaces to disturbances is likely important to an ecosystem's ability to resist cheatgrass invasion [47, 48]. Therefore, it is only appropriate to discuss soil

properties and fungal communities in association with cheatgrass- or sagebrush dominated soils but not as the cause of or effect of shifting vegetation patterns.

In the context of the limitations discussed above and emerging hypotheses for future research, the results of this study support the hypothesis that the vertical stratification of fungal community composition in the top 8 cm of sagebrush steppe soils are reduced by cheatgrass invasion with data suggesting that at least some fungal orders become dominated by genera known to contain opportunistic saprotrophic fungi. Also, the measurements of soil C and N in combination with sequencing and GRSP data suggest that proliferation of saprotrophic fungi and loss of AMF may contribute to the increased mineralization rates and decreased soil C sequestration associated with cheatgrass invasion that have been measured previously [14,15]. Lastly, all soils examined in this study are reservoirs of undescribed fungal diversity, but especially sagebrush soils, which appear to harbor conditions that foster coexistence of fungi of greater taxonomic and perhaps functional breadth than cheatgrass soils.

Sagebrush has a well-known association with AMF [49] and Hawkes et al. [18] documented a decline in AMF associated with native plant roots as a consequence of cheatgrass invasion. Although cheatgrass is colonized by AMF throughout its life cycle, colonization rates vary with season and never reach levels found in obligate mycorrhizal plants [50]. Because cheatgrass senesces in early summer, the associated AMF must survive without a host for a substantial part of the summer/fall period. The impacts of a host-free period on AMF persistence are uncertain. Nonetheless, even transient associations with cheatgrass may partially explain why we observed similar distributions of Glomeromycota, which include AMF, in sagebrush and cheatgrass-dominated soils. GRSP, the production of which has been partly attributed to members of the Glomerales order of AMF [25], was present in higher concentrations in ST than in SB, CT and CB soils (Table 1). On the contrary, we did not detect Glomeromycota in ST or CT libraries, but detected them at comparable levels in CB and SB libraries (0.6% and 0.7% of the sequence libraries, respectively). This may be due to the fact that nuclear small subunit (SSU) rRNA gene is the most commonly utilized gene for identification and phylogenetic analyses of AMF [51] and the reference databases are more robust for SSU than for the LSU gene used in this study. Additionally, we only examined classifications in the RDP database if they were $\geq$ 80% confident; there were sequences classified as Glomeromycota in the CT and ST libraries, but the classifications were below this confidence threshold indicating poor matches to the database. In future studies, SSU sequencing may be able to provide better insights into the distribution of Glomeromycota. Furthermore, activity assays would also allow one to differentiate between active and persisting AMF in soils. Nonetheless, it is interesting to note that GRSP mirrored soil C measurements in this study and that GRSP has been correlated broadly with macroaggregate formation and soil C sequestration in previous studies (Table 1; [52–53]).

The relative total soil C and N contents determined in this study are consistent with previous findings that cheatgrass-dominated soils can contain significantly reduced levels of organic matter compared to soils dominated by native vegetation [14, 54]. This pattern has been attributed to cheatgrass's allocation of more resources into aboveground production and its very shallow, fine root system relative to native perennials [14]. During active growth as well as senescence, cheatgrass introduces large amounts of labile carbon into the soil matrix, which increases decomposition rates in shallow surface layers [14]. Decomposition of labile inputs might contribute to a priming effect (e.g. [55]) and faster decay of soil organic matter than might otherwise occur in soils with native vegetation [14, 54]. Norton et al. [15] determined that soils beneath cheatgrass contained 8% more labile C than soils beneath sagebrush and C mineralization rates were 36% higher in the former than in the latter.

Fluctuations in soil water status associated with the annual cheatgrass cycle may also contribute to the faster decay of organic matter than in soils associated with native species.

Previous research has demonstrated that cheatgrass roots can elongate faster than some perennial species (e.g. *Agropyron spicatum*), allowing cheatgrass to effectively compete for moisture during the growing season [56]. However, after cheatgrass senesces and is no longer competing for moisture, these soils can retain more water; this is likely a contributing factor to our observing higher soil water contents in cheatgrass relative to sagebrush soils at the time of our sampling (September 2011), especially since cheatgrass roots are most dense in the upper 30 cm of the soil profile [57]. As water is a primary factor limiting microbial activity in arid ecosystems (e.g. [58]), periods of increased water availability combined with the increased C inputs due to the cheatgrass annual cycle may provide suitable conditions to enhance the growth of saprotrophic members of the soil microbial communities. This, in turn, may also contribute to the increased microbial activity and decomposition rates relative to that in sagebrush-dominated soils that have been observed in previous studies [14]. Norton et al. [13] demonstrated that drying-wetting cycles that occur in the summer enhanced C-mineralization in cheatgrass-invaded soils relative to those colonized by the native perennial western wheatgrass (*Pascopyrum smithii*), indicating that saprotrophic microbial populations in cheatgrass can be quickly activated when water is not limiting. Although further work is needed to directly link soil fungal community composition and function in our field site, we were able to identify compositional and structural shifts in fungal communities supporting the hypothesis that conditions in cheatgrass soils may harbor greater niche space for opportunistic saprotrophs. This is discussed in greater detail below.

Within the Helotiales and Hypocreales, the number of genera recovered in the CB composite library was nearly three times less than the number of genera present in the SB composite library (Table 4), with only one or two genera comprising the majority of the sequences in the CB composite library (S4 and S5 Tables). Interestingly, the dominant genera in cheatgrass composite libraries within these orders tended to be genera that are well-known to contain saprotrophic and also pathogenic species. For example, *Gibberella*, which harbors corn, wheat and barley pathogens [59], comprised 52.0% of the Hypocreales sequences in CB, but only 8.35% of sequences in SB. Although numbers of genera recovered within the Helotiales and Hypocreales did not differ substantially between the CT and ST composite libraries, similar shifts towards saprotroph and/or pathogen dominance was observed; *Gibberella* comprised 98.7% of the sequences in CT, but only 36.9% of the sequences in ST. Likewise, *Sclerotinia* (Helotiales), which contains necrotrophic pathogens [60], was the dominant genus within the Helotiales sequences in the CT composite library (46.0%). Other potential pathogen-containing Hypocreales genera detected in the cheatgrass libraries include *Nectria* (fruit tree parasites) and *Neonectria* (root rot; [61]).

In parallel to results of DNA-based surveys of fungi in biological soil crust and rhizosphere soils in the arid grasslands of the Sevilleta National Wildlife Refuge (SNWR; 62), Pleosporales was the most frequently recovered order across our 24 sequence libraries (Table 3). Pleosporales comprised about 50% of sequences recovered from soils in the SNWR with most OTU's classifying within the Pleosporaceae and Phaeosphaeraceae [62], the latter of which includes the genus *Phaeosphaeria*. This genus was the most frequently recovered genus within the composite CT, CB and ST libraries, but was not detected in the SB composite library. This genus is saprotrophic and contains economically important grass and cereal pathogens [63].

Broad functional classifications (e.g. saprotroph, pathogen) are not often conserved within taxonomic groups and a single organism may fit into multiple functional classifications, adjusting its role in the ecosystem depending on the prevailing environmental conditions (e.g. presence of host, availability of biogeochemical resources). Nonetheless, in shaping hypotheses for future investigation, it is interesting to note the potential for cheatgrass soils to harbor pathogenic fungal taxa, as described above. Additionally, Meyer and colleagues [64, 65] have

identified specific indigenous fungal pathogens that infect cheatgrass seedlings or seeds, which include a generalized grass pathogen and member of the Pleosporales, *Pyrenophora semeniperda*. This pathogen efficiently infects and kills cheatgrass seeds. It is interesting to note that *Pyrenophora* was only detected in the CT libraries in our study (S3 Table) fitting well with the influence of infected seeds on the composition of surface soils. Given that our data correspond well the with the expected presence of at least one pathogen containing genus (*Pyrenophora*) and that several other pathogen containing genera are more abundant in cheatgrass soils than in sagebrush soils, the potential for cheatgrass to increase the reservoir size for fungal pathogens as it invades sagebrush steppe is worth investigating in the future.

However, it should be noted that much remains to be learned about the diversity and function of Pleosporales *in situ*. Although it is the largest order within the Dothideomycetes, it has been poorly studied because of the emphasis on economically important crop pathogens within the order (e.g. [66]), which may comprise a relatively small fraction of it. It is notable that in addition to being the most abundant order recovered from the SNWR soils, the order also contained the largest number of novel sequences and lead Porras-Alfaro et al. [67] to propose arid grasslands as a "hotspot for Pleosporalean diversity". In this study, composite libraries indicate that this may also be true for sagebrush steppe soils with 56.8 to 72.9% of sequences within this order being unclassified at the genus level (S3 Table). It is interesting to note that root-associated fungi of *Bouteloua gracilis*, an aridland grass species, were dominated by a novel clade of dark septate fungi within the Pleosporales [67] and that dark septate fungi have also been found in association with cheatgrass roots [22]. Root-associated Pleosporales may contribute to the novel diversity in soils at our study as well as to C-turnover as they assume saprotrophic roles when their annual cheatgrass host senesces each year [68].

While OTU-based richness did not differ statistically across the four soil intervals examined, the classified OTUs from sagebrush-dominated soils covered a greater taxonomic breadth, with nine more orders being detected in sagebrush soils than in cheatgrass soils. Given that the fungal 18S rRNA gene copy number, a proxy for fungal biomass, did not differ statistically across soil intervals, this suggests that the size of the fungal populations in cheatgrass and sagebrush-dominated soils may be similar, but conditions in the latter may harbor a greater number of ecological niches that promote coexistence of fungi across a wide taxonomic, and perhaps, functional breadth. This is consistent with findings of Busby et al. [19] with regards to AMF, which revealed greater AMF richness associated with sagebrush than with cheatgrass roots; their findings also demonstrated that DNA sequences detected in cheatgrass roots were more phylogenetically dispersed than DNA sequences detected in sagebrush roots. They suggested that such phylogenetic dispersion might indicate competition for resources, while greater clustering of sequences associated with sagebrush roots may indicate habitat filtering or consistent selection for specific AMF species by individual shrubs. In light of this, it is interesting to highlight the relative degree of clustering of SB libraries relative to those of the other soil fractions (Fig. 2), which supports this explanation for the distribution of overall fungal communities in addition to AMF.

While functionality inferred by taxonomic affiliation suggests that cheatgrass-dominated soils may be dominated by saprotrophic taxa, we observed statistically significant increases in the relative abundance of fungal orders in sagebrush-dominated soils which are known to contain, basal, mycorrhizal and lichenized fungal lineages (Pezizales, Cantharellales, Teloschistales, Spizellomycetales). For example, interspaces around sagebrush shrubs and trunk surfaces of shrubs, harbor biological soil crusts and lichens. This would explain the relative abundance of the Teloschistales being statistically greater in ST than in CT composite libraries. Teloschistales include lichen-forming fungi [69] and their greater abundance in ST can likely be explained by the localized abundance of Teloschistales in lichenized form on sagebrush trunks, on decaying

sagebrush debris and as members of biological soil crusts. Differences in Teloschistales abundance may have implications for restoring lichenized biotic crust at invaded sites; these crusts are a primary source of N inputs in cold desert ecosystems [70].

Sagebrush also appears to provide a unique niche that is suitable for Spizellomycetales, an order within the phylum Chytridiomycota (chytrids). This order was most abundant in the composite SB libraries than in the others and by a strikingly large margin (Fig. 3). Chytrids require an aquatic habitat to complete their life cycle, and were thought to be strictly aquatic until relatively recently. Schmidt et al. [71] have documented the presence of Chytridiomycota, and Spizellomycetales in particular, in high elevation soils where melting snow pack temporarily forms aquatic environments that support abundant and active cyanobacterial and algal populations on which chytrids can feed. Although further study is needed to determine why sagebrush soils provide niche space for chytrids, it is interesting to note that conditions analogous to high elevation soils occur in sagebrush steppe; the soils are very dry most of the year, but likely saturate for short periods in the spring as snow melts offering potentially favorable conditions for chytrid growth. Chytrids have also been observed in the biotic crusts of arid soils, which might at times also harbor adequate water for growth [62]. Moss mats, which are abundant around the base of sagebrush trunks at our study site, but are sparser in cheatgrass invaded sites, might provide an additional habitat suitable for chytrids [72].

In summary, we present the first deep sequencing analysis of soil fungal community composition and structure in a cheatgrass-invaded sagebrush steppe ecosystem. This dataset provides the foundation for formulating hypotheses regarding functional shifts that are occurring in this ecosystem and the mechanisms that might be driving them. Our data support the notion that increased labile C-inputs from cheatgrass may provide an opportunity for saprotrophic fungi to compete successfully and contribute to increased C mineralization rates in cheatgrass-invaded ecosystems that have been observed previously [14, 15, 54]. However, an increased understanding of the functional roles and the seemingly large reservoir of novel fungal diversity in these ecosystems is needed to help us understand the consequences of cheatgrass invasion on ecosystem processes.

Supporting Information

S1 Fig. Normalized rarefaction curves (7,000 sequences) for each of the sequence libraries. OTU's defined at a maximum distance of 0.03. Left panel contains rarefaction curves for the 0–4 cm depth intervals and right panel contains rarefaction curves from the 4–8 cm depth intervals. Sagebrush soils (●); cheatgrass soils (○).
(TIFF)

S2 Fig. Rank abundance curves for the 50 most abundant OTU's in each of the soil fractions. Data for each rank is the average number of sequences in that rank (n = 6 standard error).
(TIFF)

S1 Table. The total number of sequences in each library, the number of sequences that classified within the fungal domain (with 100% confidence) as well as at the phylum, class and order levels with $\geq$80% confidence.
(DOCX)

S2 Table. Genus-level composition (%) of sequences classifying within the Agaricales for composite libraries from each of the four soil intervals examined. Composite libraries for each of the four soil intervals include the libraries generated from the six field replicates.
(DOCX)

S3 Table. Genus-level composition (%) of sequences classifying within the Pleosporales for composite libraries from each of the four soil intervals examined. Composite libraries for each of the four soil intervals include the libraries generated from the six field replicates. (DOCX)

S4 Table. Genus-level composition (%) of sequences classifying within the Hypocreales for composite libraries from each of the four soil intervals examined. Composite libraries for each of the four soil intervals include the libraries generated from the six field replicates. (DOCX)

S5 Table. Genus-level composition (%) of sequences classifying within the Helotiales for composite libraries from each of the four soil intervals examined. Composite libraries for each of the four soil intervals include the libraries generated from the six field replicates. (DOCX)

Acknowledgments

We thank J.S. Lockhart and J.T. Werth for excellent technical support and Dr. R.S. Inouye for orientation and access to the Barton Road Ecological Research Area.

Author Contributions

Conceived and designed the experiments: CFW GMK. Performed the experiments: CFW GMK. Analyzed the data: CFW GMK KA. Contributed reagents/materials/analysis tools: CFW GMK KA. Wrote the paper: CFW GMK KA.

References

1. Wisdom MJ, Rowland MM, Suring LH, Schueck L, Meinke CW et al. (2005) Evaluating species of conservation concern at regional scales. Chap. 1 in part I: Methods of regional assessment for sagebrush-associated species of conservation concern, Pages 5–74 In: Wisdom MJ, Rowland MM, Suring LH (eds.). Habitat Threats in the Sagebrush Ecosystem: Methods of regional assessment and applications in the Great Basin. Alliance Communications Group. Lawrence, KS: 5.
2. Bradley BA, Mustard JF (2005) Identifying land cover variability distinct from land cover change: cheatgrass in the Great Basin. Rem Sens Environ 94: 204–213.
3. Knapp PA (1996) Cheatgrass (*Bromus tectorum L.*) dominance in the Great Basin Desert: History, persistence, and influences to human activities. Glob Env Change 6: 37–52.
4. West NE (1999) Synecology and disturbance regimes of sagebrush steppe ecosystems. In: Entwistle PG, DeBolt AM, Kaltenecker JH, Steenhof K (Eds.), Sagebrush Steppe Ecosystems Symposium. Publication No. BLM/ID/PT-001001+1150, Bureau of Land Management, Boise State University, Boise, ID.
5. D'Antonio CM, Vitousek PM (1992) Biological invasions by exotic grasses, the grass/fire cycle, and global change. Ann Rev Ecol Sys 23: 63–87.
6. Whisenant SG (1990) Changing fire frequencies on Idaho's Snake River Plains: ecological and management implications. In: McArthur DE, Romney EM, Smith SD, Tueller PT (Eds.), Proceedings of the symposium on cheatgrass invasion, shrub die-off, and other aspects of shrub biology and management, Las Vegas, NV, April 5–7, 1989, USDA Forest Service, Intermountain Research Station, Ogden, UT, GTR-INT-276, pp. 4–10.
7. Welch BL (2005) Big sagebrush: a sea fragmented into lakes, ponds, and puddles. Vol. General Technical Report RMRS-GTR-144: U. S. Department of Agriculture Forest Service, Rocky Mountain Research Station.
8. Boyle SA, Reeder DR (2005) Colorado sagebrush: a conservation assessment and strategy. Grand Junction: Colorado Division of Wildlife.
9. Pellant M (1996) Cheatgrasss: The invader that won the West. Bureau of Land Management, Idaho State Office. Interior Columbia Basin Ecosystem Management Project.

10. Belnap J (1994) Potential role of cryptobiotic crusts in semiarid rangelands. In: Ecology and management of annual rangelands, Monsen SB and Kitchen SG: USDA Forest Service Technical Report INT-GTR-313. Intermountain Research Station, Ogden, UT.
11. Evans RD, Rimer R, Sperry L, Belnap J (2001) Exotic plant invasion alters nitrogen dynamics in an arid grassland. Ecol Apps 11: 1259–1260.
12. Rau BM, Johnson DW, Blank RR, Lucchesi A, Caldwell TG et al. (2011) Transition from sagebrush steppe to annual grass (*Bromus tectorum*): influence on belowground carbon and nitrogen. Rangeland Ecol Manage 64: 139–147.
13. Norton U, Mosier AR, Morgan JA, Derner JD, Ingram LJ et al. (2008) Moisture pulses, trace gas emission and soil C and N in cheatgrass and native grass-dominated sagebrush-steppe in Wyoming, USA. Soil Biol Biochem 40: 1421–1431.
14. Norton JB, Monaco TA, Norton JM,Johnson DA, Jones TA (2004) Cheatgrass invasion alters soil morphology and organic matter dynamics in big sagebrush-steppe rangelands. USDA Forest Service Proceedings RMRS-P-31.
15. Norton U, Saetre P, Hooker TD, Stark JM (2012) Vegetation and moisture controls on soil carbon mineralization in semiarid environments. Soil Sci Soc Am J. 76: 1038–1047.
16. Kuske CR, Ticknor LO, Miller ME, Dunbar JM, Davis JA et al. (2002) Comparison of soil bacterial communities in rhizosphere of three plant species and the interspaces in an arid grassland. Appl Environ Microbiol 68: 1854–1863. PMID: 11916705
17. Belnap J, Phillips SL (2001) Soil biota in an ungrazed grassland: response to annual grass (Bromus tectorum) invasion. Eco Apps 11: 1261–1275.
18. Hawkes CV, Belnap J, D'Antonio C, Firestone MK (2006) Arbuscular mycorrhizal assemblages in native plant roots change in the presence of invasive exotic grasses. Plant Soil 281: 369–380.
19. Busby RR, Stromberger ME, Rodriguez G, Gebhart DL, Paschke MW (2013) Arbuscular mycorrhizal fungal community differs between a coexisting native shrub and introduced annual grass. Mycorrhiza 23: 129–141. doi: 10.1007/s00572-012-0455-x PMID: 22864708
20. Carter KA, Smith JF, White MM, Serpe MD (2014) Assessing the diversity of arbuscular mycorrhizal fungi in semiarid shrublands dominated by *Artemisia tridentate* ssp. *Wyomingensis*. Mycorrhiza. 24: 301–314. doi: 10.1007/s00572-013-0537-4 PMID: 24249492
21. Duke SE, Jackson RB, Caldwell MM (1994) Local reduction of mycorrhizal arbuscule frequency in enriched soil microsites. Can J Bot 72: 998–1001.
22. Goldberry S, Deckert R (2006) Comparison of mycorrhizal colonization frequency of *Bromus tectorum* and native grass species at Antelope Island State Park. NCUR 20, University of North Carolina- Asheville, NC. 20th National Conference on Undergraduate Research.
23. Rillig MC, Mardatin NF, Leifheit EF Antunes PM (2010) Mycelium of arbuscular mycorrhizal fungi increases soil water repellency an is sufficient to maintain water-stable soil aggregates. Soil Biol Biochem 42: 1189–1191.
24. Wilson GWT, Rice C, Rillig MC, Springer A, Hartnett D (2009) Soil aggregation and carbon sequestration are tightly correlated with the abundance of arbuscular mycorrhizal fungi: results from long-term field experiments. Ecol Lett 12: 452–461. doi: 10.1111/j.1461-0248.2009.01303.x PMID: 19320689
25. Rillig MC (2004) Arbuscular mycorrhizae, glomalin, and soil aggregation. 84: Can J Soil Sci 355–363.
26. Lindahl BD, de Boer W, Finlay RD (2010) Disruption of root carbon transport into forest humus stimulates fungal opportunists at the expense of mycorrhizal fungi. The ISME J 4: 872–881. doi: 10.1038/ismej.2010.19 PMID: 20220789
27. Bardgett RD, Freeman C, Ostle NJ (2008) Microbial contributions to climate change through carbon cycle feedbacks. The ISME J 2: 805–814. doi: 10.1038/ismej.2008.58 PMID: 18615117
28. King GM (2011) Enhancing soil carbon storage for carbon remediation: potential contributions and constraints by microbes. Trends Microbiol 19: 75–84. doi: 10.1016/j.tim.2010.11.006 PMID: 21167717
29. van der Putten W, Klironomos JN, Wardle DA (2007) Microbial ecology of biological invasions. The ISME J 1: 28–37. PMID: 18043611
30. Inouye RS (2002) Sampling effort and vegetative cover estimates in sagebrush steppe. Western North American Naturalist 62: 360–364.
31. Inouye RS (2006) Effects of shrub removal and nitrogen addition on soil moisture in sagebrush steppe. J Arid Environ 65: 604–618.
32. Blight GE, Leong EC (2012) Mechanics of Residual Soils. Second Edition. CRC Press. Taylor and Francis Group, London, UK. p. 50. Chapter 2. Pp. 41–60. Microstructure, mineralogy and classification of residual soils. Fourie AB, Irfan TY, Queiroz de Carvalho JB, Simmons JV, Wesley LD. doi: 10.3390/jpm2020050 PMID: 25562799

33. Harris D, Horwáth WR, van Kessel C (2001) Acid fumigation of soils to remove carbonates prior to total organic carbon or CARBON-13 isotopic analysis. Soil Sci Soc Am J 65:1853–1856.

34. Bedini S, Pellegrino E, Avio L, Pellegrini S, Bazzoffi P, et al. (2009) Changes in soil aggregation and glomalin-related soil protein content as affected by the arbuscular mycorrhizal fungal species *Glomus mosseae* and *Glomus intraradices*. Soil Biol Biochem 41:1491–1496.

35. Sigma-Aldrich Co. LLC (2013) Bradford Reagent Technical Bulletin. Catalog Number B6916.

36. Aho K (2014) Foundational and Applied Statistics for Biologists Using R. CRC Press. PMID: 25566584

37. Brunner E, Domhof S, Langer F (2002) Nonparametric Analysis of Longitudinal Data in Factorial Experiments. New York: Wiley. PMID: 25057650

38. Wilcox RR (2005) "Comparing Multiple Dependent Groups in: Introduction to robust estimation and hypothesis testing. Burlington, MA: Elsevier Academic Press Publications. pp. 369–375. PMID: 16676562

39. R Development Core Team (2011) R: A language and environment for statistical computing, Vienna, Austria.

40. Weber CF, Vilgalys R, Kuske CR (2013) Changes in fungal community composition in response to elevated atmospheric CO_2 and nitrogen fertilization varies with soil horizon. Front Microbiol.

41. Castro HF, Classen AT, Austin EE, Norby RJ, Schadt CW (2010) Soil Microbial community responses to multiple experimental climate change drivers. Appl Environ Microbiol 76: 99–1007.

42. Schloss PD, Wescott SL, Ryabin T, Hall JR, Hartman M, et al. (2009) Introducing Mothur: Open Source, Platform-independent, Community-supported Software for Describing and Comparing Microbial Communities. Appl Environ Microbiol 75:7537–7541. doi: 10.1128/AEM.01541-09 PMID: 19801464

43. Liu KL, Porras-Alfaro A, Kuske CR, Eichorst SA, Xie G. (2012) Accurate, rapid taxonomic classification of fungal large-subunit rRNA genes. Appl Environ Microbiol 71: 1523–1533.

44. Bray RJ, Curtis JT (1957) An ordination of upland forest communities of Southern Wisconsin. Ecol. Mong. 27: 325–349.

45. Oksanen J, Blanchet FG, Kindt R, Legendre P, Minchin PR et al. (2013) vegan: Community Ecology Package. R package version 2.0–10. Available: http://CRAN.R-project.org/package = vegan

46. Chambers JC, Roundy BA, Blank RR, Meyer SE, Whittaker A (2007) What makes Great Basin sagebrush ecosystems invasible by *Bromus tectorum* ? Ecol Monog 77: 117–145.

47. Hoover AN, Germino MJ (2012) A common-garden study of resource-island effects on a native and an exotic, annual grass after fire. Rangeland Ecol Manage 65: 160–170.

48. Sankey JB, Germino MJ, Sankey TT, Hoover AN (2012) Fire effects on the spatial patterning of soil properties in sagebrush steppe, USA: a meta-analysis Int J Wildland Fire 21: 545–556.

49. Klironomos JN, Ursic M, Rillig M, Allen MF (1998) Interspecific differences in the response of arbuscular mycorrhizal fungi to *Artemisia tridentata* grown under elevated atmospheric CO_2. New Phytol 4: 599–605.

50. Busby RR, Passchke MW, Stromberger ME, Gebhart DL (2012) Seasonal variation in arbuscular mycorrhizal fungi root colonization of cheatgrass (*Bromus tectorum*), an invasive winter annual. J Ecosys Ecog S8.

51. Krüger M, Krüger C, Walker C, Stockinger H, Schüßler A (2011) Phylogenetic reference data for systematics and phylotaxonomy of arbuscular mycorrhizal fungi from phylum to species level. New Phyt 193:970–984.

52. Rillig MC, Steinberg PD (2002) Glomalin production by an arbuscular mycorrhizal fungus: a mechanism of habitat modification? Soil Biol Biochem 34: 1371–1374.

53. Rillig MC, Ramsey PW, Morris S, Paul EA (2003) Glomalin, an arbuscular-mycorrhizal fungal soil protein, responds to land-use change. Plant Soil 253: 293–299.

54. Sperry LJ, Belnap J, Evans RD (2006) *Bromus tectorum* invasion alters nitrogen dynamics in an undsturbed arid grassland ecosystem. Ecology 87: 603–615. PMID: 16602290

55. Sébastien F, Bardoux G, Abbadie L, Mariotti A (2004) Carbon input to soil may decrease soil carbon content. Ecol Lett 7: 314–320

56. Harris GA, Wilson AM (1970) Competition for moisture among seedlings of annual and perennial grasses as influenced by root elongation at low temperature. Ecology. 51(3): 530–534.

57. Hulbert LC (1955) Ecological studies of Bromus tectorum and Other Annual Bromegrasses. Ecol Monog 25(2): 181–213.

58. Cregger MA, Schadt CW, McDowell NG, Pockman WT, Classen AT (2012) Responses of the soil microbial community to changes in precipitation in a semiarid ecosystem. Appl Env Microbiol 78(24): 8587. doi: 10.1128/AEM.02050-12 PMID: 23023755

59. Leslie JF, Summerell BA (2006) The *Fusarium* Laboratory Manual. Blackwell Professional. Ames, IA.

60. Amselem J, Cuomo CA, van Kan JAL, Viaud M, Benito EP et al. (2011) Genomic analysis of the necrotrophic fungal pathogens *Sclerotinia sclerotiorum* and *Botryotinia cinerea*. PLoS Genetics 7(8): e1002230. doi: 10.1371/journal.pgen.1002230 PMID: 21876677

61. Menkis A, Burokiene D (2012) Distribution and genetic diversity of the root-rot pathogen *Neonectria macrodidyma* in a forest nursery. Forest Path 42:79–83.

62. Porras-Alfaro A, Herrera J, Natvig DO, Lipinski K Sinsabaugh R (2011) Diversity and distribution of soil fungal communities in a semiarid grassland. Mycologia 103: 10–21. doi: 10.3852/09-297 PMID: 20943560

63. McDonald MC, Oliver RP, Friesen TL, Brunner PC, McDonald BA (2013) Global diversity and distribution of three necrotrophic effectors in *Phaeosphaeria nodorum* and related species. New Phytol 199: 241–251. doi: 10.1111/nph.12257 PMID: 23550706

64. Baughman OW, Meyer SE (2013) Is *Pyrenophora semeniperda* the cause of downy brome (*Bromus tectorum*) die-offs? Invasive Plant Sci Manage 6:105–111.

65. Meyer SE, Nelson DL, Clement S, Beckstead J (2008) Cheatgrass (*Bromus tectorum*) biocontrol using indigenous fungal pathogens. USDA Forest Service Proceedings RMRS-P-52.2008.

66. Ohm RA, Feau N, Henrissat B, Schoch CL, Horwitz BA et al. (2012) Diverse lifestyles and strategies of plant pathogenesis encoded in the genomes of eighteen *Dothideomycetes* fungi. PLoS Pathog 8(12): e1003037. doi: 10.1371/journal.ppat.1003037 PMID: 23236275

67. Porras-Alfaro A, Herrera J, Sinsabaugh RL, Odenbach KJ, Lowrey T et al. (2008) Novel root fungal consortium associated with a dominant desert grass. Appl Environ Microbiol 74: 2805–2813. doi: 10.1128/AEM.02769-07 PMID: 18344349

68. Promputtha I, Lumyong S, Dhanasekaran V, Huge E, McKenzie C et al. (2007) A phylogenetic evaluation of whether endophytes become saprotrophs at host senescence. Microb Ecol 53: 579–590. PMID: 17410394

69. Nash TH III (2008) Lichen Biology Second Edition, Cambridge University Press, Cambridge, UK. Stress, Physiology and the Symbiosis. Beckett, RP, Kranner I and Minibayeva FV. 134–180. doi: 10.1161/CIRCOUTCOMES.108.825232 PMID: 20031801

70. Evans RD, Ehleringer JR (1993) Broken nitrogen cycles in arid lands? Evidence from $\delta^{15}N$ of soils. Oecologia 94: 314–317.

71. Schmidt SK, Naff CS, Lynch RC (2011) Fungal communities at the edge: ecological lessons from high alpine fungi. Fungal Ecol 5:443–452.

72. Letcher PM, Powell MJ (2002) Frequency and distribution patterns of zoosporic fungi from moss-covered and exposed forest soils. Mycologia 94: 761–771. PMID: 21156550
